# Activation of the human insulin receptor by non-insulin-related peptides

**DOI:** 10.1038/s41467-022-33315-8

**Published:** 2022-09-28

**Authors:** Nicholas S. Kirk, Qi Chen, Yingzhe Ginger Wu, Anastasia L. Asante, Haitao Hu, Juan F. Espinosa, Francisco Martínez-Olid, Mai B. Margetts, Faiz A. Mohammed, Vladislav V. Kiselyov, David G. Barrett, Michael C. Lawrence

**Affiliations:** 1 WEHI, 1G Royal Parade, Parkville, VIC 3052 Australia; 2 Department of Medical Biology, Faculty of Medicine, Dentistry and Health Sciences, University of Melbourne, Parkville, VIC 3050 Australia; 3 Eli Lilly and Company, Lilly Corporate Center, Indianapolis, IN 46285 USA; 4 Advanced Testing Laboratories, Blue Ash, OH 45242 USA; 5 Centro de Investigación Lilly S.A., Avda. de la Industria 30, Alcobendas, Madrid 28108 Spain

**Keywords:** Cryoelectron microscopy, Kinases

## Abstract

The human insulin receptor signalling system plays a critical role in glucose homeostasis. Insulin binding brings about extensive conformational change in the receptor extracellular region that in turn effects trans-activation of the intracellular tyrosine kinase domains and downstream signalling. Of particular therapeutic interest is whether insulin receptor signalling can be replicated by molecules other than insulin. Here, we present single-particle cryoEM structures that show how a 33-mer polypeptide unrelated to insulin can cross-link two sites on the receptor surface and direct the receptor into a signalling-active conformation. The 33-mer polypeptide engages the receptor by two helical binding motifs that are each potentially mimicable by small molecules. The resultant conformation of the receptor is distinct from—but related to—those in extant three-dimensional structures of the insulin-complexed receptor. Our findings thus illuminate unexplored pathways for controlling the signalling of the insulin receptor as well as opportunities for development of insulin mimetics.

## Abstract (web summary)

The regulation of plasma glucose levels is effected by insulin. Here, the authors reveal atomic detail of how peptides distinct from insulin bind to and activate the insulin receptor, with implications for design of small-molecule insulin mimetics.

## Introduction

**Fig. 1:**
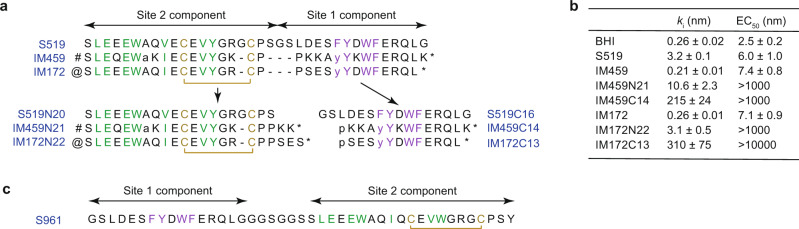
IR-binding peptides and their biochemical properties. **a** [Site-2]-[Site-1] fusion peptides S519^6^, IM459 and IM172^7^ and their isolated [Site 2]- and [Site 1] components. Non-native amino acids and modifications are as follows:- a: 2-aminoisobutyric acid; p: N-acetylproline; y: O-methyltyrosine; #: N-terminal (4-aminomethyl)phenylacetylation; @: N-terminal phenylacetylation; *: C-terminal amidation. **b** Receptor inhibition (*k*_i_) and auto-phosphorylation (EC_50_) constants (see Supplementary Fig. 2b–f). BHI = biosynthetic human insulin (control). Errors are SEM; *n* = 3 independent replicates. Source data are provided as a Source Data file. **c** The [Site-1]-[Site-2] fusion peptide S961^15^. Individual residues colored green and mauve within panels a and c are those that are part of the defining motifs of the [Site 2]- and [Site 1] peptides, respectively (see Supplementary Fig. 1); residues involved in disulfide bridge formation are colored in yellow, with the corresponding bridge shown as yellow square bracket.

The human insulin receptor (hIR) is an [αβ]_2_ homodimeric receptor tyrosine kinase that upon insulin binding signals into metabolic pathways that effect glucose homeostasis^1–5^. Of intense interest in the treatment of diabetes is the development of biologics or small molecules that have improved pharmacodynamic and pharmacokinetic properties with respect to native insulin. In particular, a family of non-insulin-related peptides has been reported that in animal models can both activate the insulin receptor pathway and lower blood glucose levels^6,7^. These peptides are themselves N- to C-terminal fusions of respective “Site 2” and “Site 1” peptides derived originally from a phage display study^8^. The Site 1/Site 2 nomenclature for these peptides arose originally from partitioning the peptides into non-competing groups and did not initially imply any correspondence to what are termed the respective binding sites 1 and 2 of insulin on the hIR surface^9^. Site 2 peptides were characterized initially as containing a disulfide loop and Site 1 peptides as containing the penta-motif FYXWF (X = any amino acid)^8^—these motifs were subsequently refined as the so-called “D8” and “A6” motifs, respectively (Supplementary Fig. 1a)^10^. Site 1 peptides bind to domain L1 of hIR, mimicking the interaction of the αCT segment of hIR with that domain^11,12^ (see Supplementary Fig. 2a for hIR domain nomenclature). Recently, a [Site 2] peptide “S592” (sequence SLEEEWAQIECEVWGRGCPSY^13^) has been shown to bind competitively to insulin’s site 2 (located on receptor domain FnIII-1 or, equivalently, FnIII-1′)^14^. Whereas biological activity of both Site 1 and Site 2 peptides in isolation is minimal^6^, [Site 2]-[Site 1] fusion peptides are IR agonists, with the most potent reported being S519 (Fig. 1a, b)^6^. [Site 1]-[Site 2] fusion peptides are, in contrast, receptor antagonists, an exemplar being S961 (Fig. 1c)^6,15^. Development of the above peptide family has, however, been stalled due to lack of knowledge of how the [Site 2]-[Site 1] fusion peptides bind to and direct hIR into a signalling-active conformation.

Here, we describe two [Site 2]-[Site 1] fusion peptides: IM459 and IM172^7^ (the prefix “IM” being an abbreviation for “insulin mimetic”, with their respective N- and C-terminal fragments being denoted by the suffix “N” or “C” followed in turn by the number of residues within the fragment; see Fig. 1a, b). We show, using single-particle cryo-electron microscopy (“cryoEM”), that IM459 cross-links hIR domain L1 to hIR domain FnIII-1′ and, in so doing, releases constraints on the membrane-proximal hIR domain pairs [FnIII-2]-[FnIII-3] and [FnIII-2′]-[FnIII-3′]. To obtain a high-resolution view of how the fusion peptides interact with domain FnIII-1′, we determined a 2.9 Å resolution cryoEM structure of the Site 2 component (“IM172N22”) of IM172 (Fig. 1a) in complex with the hIR ectodomain. Taken together, these data illuminate pathways for the development of novel hIR agonists useful in the treatment of diabetes.

## Results

### Biochemical properties of the peptides

The fusion peptides IM172^7^ and IM459 are derivatives of the [Site 2]-[Site 1] peptide S519^6^; they contain mutations intended *inter alia* to reduce proteolytic susceptibility (Fig. 1b). IM459 and IM172 display comparable sub-nM affinity for hIR to that of biosynthetic human insulin (BHI, “Humalin®”), with IM172N22 (the Site 2 component of IM172) displaying ∼10-fold weaker affinity for hIR-A than BHI and IM172C13 (the Site 1 component of IM172; Fig. 1c) displaying ∼1000-fold weaker affinity for hIR-A than BHI (Supplementary Fig. 2b). S519, IM459 and IM172 have ∼2.5-fold weaker activity than BHI in an hIR autophosphorylation assay, whereas IM172N22 and IM172C13 (Fig. 1c) are devoid of detectable activity (Supplementary Fig. 2c). In contrast to BHI, none of the [site 2]-[site 1] peptides accelerate dissociation of pre-bound I125-insulin tracer at “infinite” dilution from native membrane-expressed hIR^16^; instead, IM459 decreases dissociation of the pre-bound insulin tracer (Supplementary Fig. 2d) and S519 and IM172 trend similarly, but more weakly (Supplementary Fig. 2e, f). IM459 can thus bind to the pre-formed hIR-insulin complex and decrease insulin dissociation from that complex. We further tested the effects of the site 2 and site 1 parts of IM459 (*i.e*., IM459N21 and IM459C14, respectively), and found that the effect of IM459 is fully reproduced by IM459N21, whereas IM459C14 had no effect on the dissociation of pre-bound insulin (Supplementary Fig. 2g, h).

### The IM459-bound hIR ectodomain

**Fig. 2:**
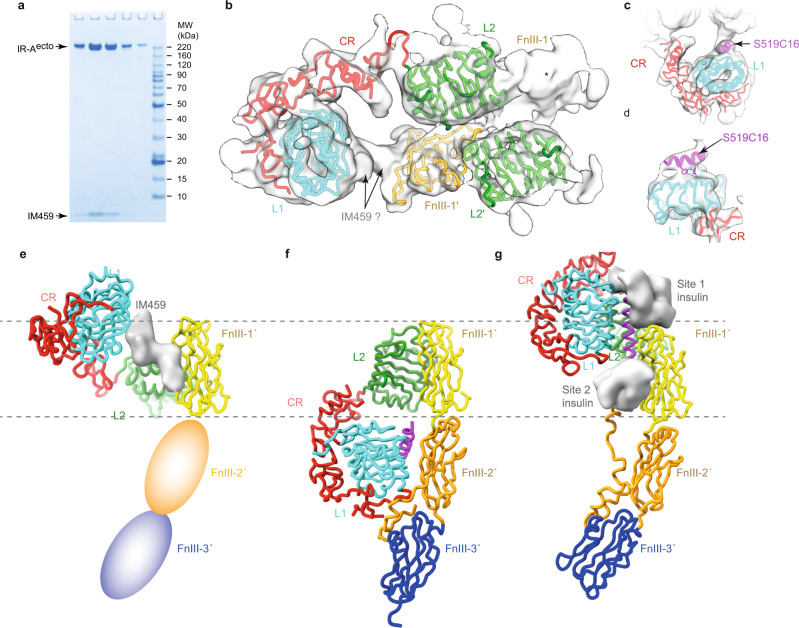
CryoEM structure of the IM459-bound hIR-A^ecto^. **a** NuPAGE 4-12% Bis*-*Tris gel (non-reducing conditions) of pooled fractions evidencing the presence of the IM459 peptide within the purified complex. This gel experiment was conducted *n* = 1 times. **b** Focus-refined cryoEM map showing the fit of IR domains L1, CR, L2, FnIII-1′, and L2′ and the residual density attributable to IM459; density corresponding to domain FnIII-1 is asterisked. **c**,**d** Map as in b, showing respective orthogonal views of the rigid-body fit of the co-complex of S519C16 and the hIR L1-CR module (PDB entry 5J3H)^12^. **e-g** Comparison of the respective half-structures of the IM459-bound hIR-A^ecto^, the apo hIR ectodomain (PDB entry 4ZXB), and the four-insulin-bound hIR ectodomain (PDB entry 6SOF), displayed with a common alignment of their respective domains FnIII-1′. In e, the location of domains FnIII-2′ and FnIII-3′ are indicative only. See also Supplementary Fig. 3. In panels **b**-**g**, receptor domain colors match those in the primary structure domain layout provided in Supplementary Fig. 2a.

Our cryoEM study of the IM459-complexed insulin receptor is based on single-particle cryoEM imaging of the peptide in complex with a receptor construct termed “hIR-A^ecto^”. hIR-A^ecto^ spans the entire extracellular region of the receptor (residues 1-914; see Methods) and its sequence is based on that of isoform A of the receptor, *i.e*., that of the exon 11^-^ isoform^17^. The resultant three-dimensional (3D) map displayed pseudo-C2 symmetry, refinement with imposed C2 symmetry led to maps with ill-defined receptor domains. We thus undertook focussed refinement, masking the region corresponding to domains L1, CR, L2, FnIII-1, L2 and FnIII-1′—this process resulted in a map of *ca* 5.0 Å resolution (Fig. 2a; with detail in Methods, Supplementary Fig. 3 and Supplementary Table 1). The map was then interpretable as comprising (i) the four-domain [L2-(FnIII-1)]_2_ module of the receptor, (ii) an L1-CR module, adjacent to domain L2 and with domain L1 in proximity to domain FnIII-1′, and (iii) a considerably more mobile [L1-CR]′ module on the opposite side of the two-fold axis (Fig. 2a). Support for this interpretation was provided *inter alia* by the distinctive barrel-like nature of the density for domain L1, extended density features at known N-linked glycosylation sites^18^, and distinctive density corresponding to known α-helical secondary-structure elements within the identified domains. Two tube-like density features were then visible, sandwiched between the juxtaposed surfaces of domains L1 and FnIII-1′ (Fig. 2a). The first of these was in a location compatible with that seen for the [Site 1]-only peptide S519C16 in its co-crystal structure with an isolated L1-CR module (PDB entry 5J3H)^12^ (Fig. 2b, c), allowing putative interpretation as the Site 1 component of IM459. The second (and adjacent) tube-like density feature on domain FnIII-1′ was hence provisionally interpreted as the Site 2 component of IM459, possibly also in helical conformation.

Attempts to improve the resolution of this focus-refined structure failed, likely due to flexibility in the linker between two components of the peptide and the resultant slight mobility of domain L1 with respect to domain FnIII-1′ (Fig. 1b). The relative disorder of the [L1-CR]′ module and domain FnIII-1 with respect to the remainder of the structure may reflect individual IM459 peptides being unable to cross-link domains L1 and FnIII-1′ and domains L1′ and FnIII-1 simultaneously; alternatively, IM459 peptides may be able to simultaneously cross-link domain L1 to domain FnIII-1′ and domain L1′ to domain FnIII-1 but, in so doing, destabilize the central [L2-(FnIII-1)]_2_ module. Our maps do not distinguish between these possibilities. We also deduce that the hIR-A^ecto^ domains FnIII-2, FnIII-2′, FnIII-3, and FnIII-3′ are considerably mobile, though we note weak density for an apparent single FnIII-2 domain in some 2D particle classes (Supplementary Fig. 3a). Analogous mobility has been observed in some cryoEM structures of the insulin-bound receptor ectodomain^2^, consistent with the release of the L1-CR modules from their partner FnIII-2′ domains freeing up the membrane-proximal FnIII-3 domains to enable signal transduction^19^.

The relative conformation of the focus-refined hIR-A^ecto^ domains reflects that of the insulin-bound receptor, in that the L1-CR module is displaced away from its apo-interaction with domain FnIII-2′ and directed instead towards the receptor “head” (Fig. 2e–g). The relative positioning of domains L1 and FnIII-1′ is, however, distinct from that seen in the site 1 to site 2 cross-linked halves of the three-insulin-bound structures of hIR reported recently (Supplementary Fig. 4a–e)^14^.

### The IM172N22-bound hIR ectodomain

Attempts to obtain a cryoEM structure of the IR ectodomain in complex with the [Site 2]-peptide IM172N22 did not succeed, likely because IM172N22 binding alone fails to reduce the known conformational flexibility of the apo ectodomain^2,5,20^. We thus bound IM172N22 to the ectodomain construct IRΔβ-zip^3^ pre-complexed with insulin and with the variable domain Fv 83-7 of monoclonal antibody 83-7^21^. IRΔβ-zip spans receptor residues 1-916 of the A isoform of the receptor and has a leucine-zipper segment^22^ attached at its β-chain C terminus to add stability to the construct^3,23^. The “Δβ” mutation within this construct refers to the deletion of a short but highly glycosylated segment near the N terminus of the receptor β chain^20^. Fv 83-7 aids cryoEM particle alignment^3,12^. The epitope of Fv 83-7 lies within the cysteine-rich domain (CR) of the receptor (Supplementary Fig. 2a) and its parent antibody is known not to compete with insulin for receptor binding^21^. Critically, within the insulin + [Fv 83-7]+IRΔβ-zip co-complex (PDB entries 6HN5 and 6HN4^3^), the above-anticipated [Site 2]-peptide binding surface is exposed.

**Fig. 3:**
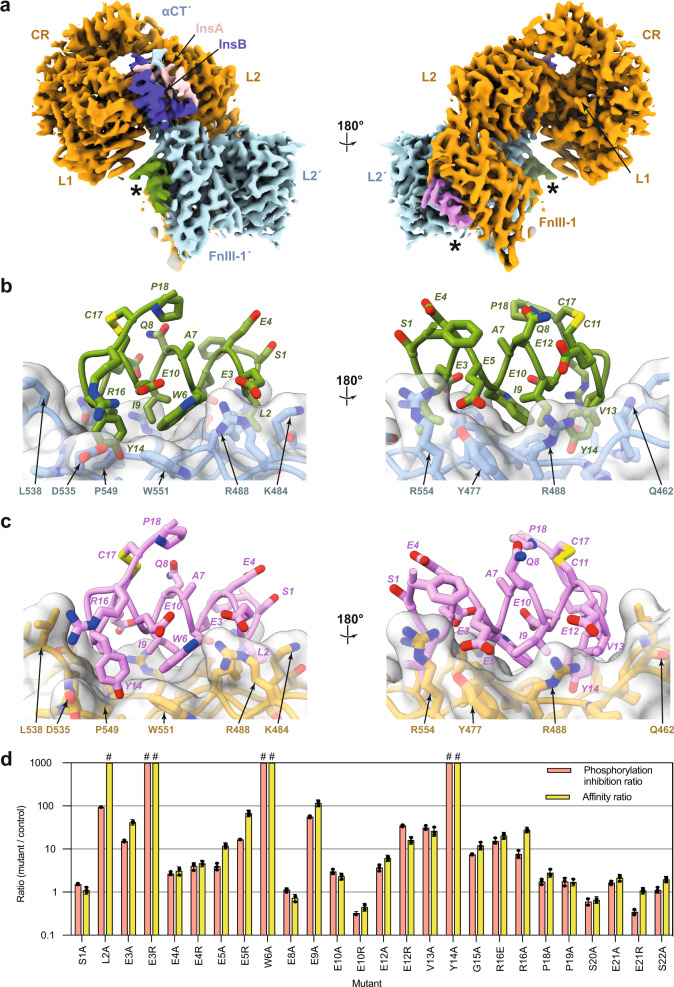
CryoEM structure of the IM172N22-complexed IR-A^ecto^ + insulin. **a** Overview of the cryoEM map associated with the receptor “head” region. Asterisks (*) indicate the two copies of IM172N22 (light magenta and green respectively). Receptor domain colors are as follows: orange, L1-CR-L2-[FnIII-1] module; cyan: L2′-[FnIII-1]′ module and αCT’ segment; pink: insulin A chain; dark blue: insulin B chain. **b** Atomic model of IM172N22 (carbon atoms green) bound to IR-A^ecto^ domain FnIII-1′ (carbon atoms light cyan). **c** Atomic model of IM172N22 (carbon atoms light magenta) bound to IR-A^ecto^ domain FnIII-1 (carbon atoms light orange). In panels **b** and **c**, oxygen atoms are in red, nitrogen atoms in blue and sulfur atoms in yellow. **d** Relative affinity ratio (*K*_i_^(mutant)^/*K*_i_^(control)^) and relative phosphorylation inhibition ratio (IC50^(mutant)^/IC50^(control)^) of mutant peptides. Mutations indicated are with respect to the control peptide (= IM172N22 devoid of N-terminal phenylacetylation). Error bars reflect standard error of the mean of each ratio, based on *n* = 3 independent technical replicates and with aberrant measurements omitted as indicated in the associated Source Data file. #: ratio >4000.

Our single-particle cryoEM map of the IM172N22 + insulin + [Fv 83-7] + IRΔβ-zip co-complex (obtained at a GS-FSC resolution of 2.9 Å; see Methods, Supplementary Fig. 5 and Supplementary Table 1 for detail) allowed ready recognition and docking of the insulin + [Fv 83-7] + IRΔβ-zip “head” module derived from PDB 6HN5^3^ (Fig. 3a). Inspection then indicated additional density features on the surfaces of domains FnIII-1 and FnIII-1′, situated in equivalent positions to that observed for the putative [Site 2]-binding region of IM459 in the structure presented above (Fig. 2a). Both these density features permitted atomic modelling of the peptide (Fig. 3b, c and Supplementary Fig. 6), despite the ambiguities associated with potential radiation damage to any or all the glutamate and cysteine residues within the peptide^24^.

The salient features of the IM172N22 FnIII-1 interaction are as follows (Fig. 3c): (i) IM172N22 residues Glu3 to Val13 adopt a mostly α-helical conformation. (ii) The IM172N22 Leu2 side chain is located within a pocket formed by the side chains of IR Arg479, Ser481, Lys484, Leu486, and Leu552, (iii) The IM172N22 Glu3 side chain is capable of an electrostatic interaction with those of IR Lys484 and Arg554. (iv) The IM172N22 Glu5 side chain stacks against the ring of the peptide’s N-terminal phenylacetyl moiety, is in electrostatic interaction with that of IR Arg479, and is capable of forming a hydrogen bond with the side-chain hydroxyl of IR Tyr477. (v) IM172N22 Trp6 docks into a hydrophobic pocket formed by IR Leu486, Gly550, Trp551, and Leu552 and is stabilized within the peptide by packing against the side chains of IM172N22 Glu10 and Tyr14. (vi) IM172N22 Ile9 docks against the side chains of IR Leu486 and Arg488. (vii) Glu12 is capable of an electrostatic interaction with Arg488. (viii) The side chains of IM172N22 Val13 and Tyr14 dock into a pocket formed by the side chains of IR Ile534, Asp535, Pro536, Pro537, Leu538, Asn547, Pro549, Gly550, and Trp489. (ix) IM172N22 Arg16 forms an electrostatic interaction with IR Asp535. (x) The side chains of IM172N22 Ser1, Glu4, Ala7, Gln8, Glu10, Cys11, Arg16, Cys17, Pro18, and Pro19 are devoid of interaction with IR. The above interactions align completely with the “D8” motif^10^ (Supplementary Fig. 1) and are effectively conserved across the two copies of IM172N22 (Fig. 3c), despite the non-equivalence of domains FnIII-1 and FnIII-1′ within the single-insulin complexed structure.

**Fig. 4:**
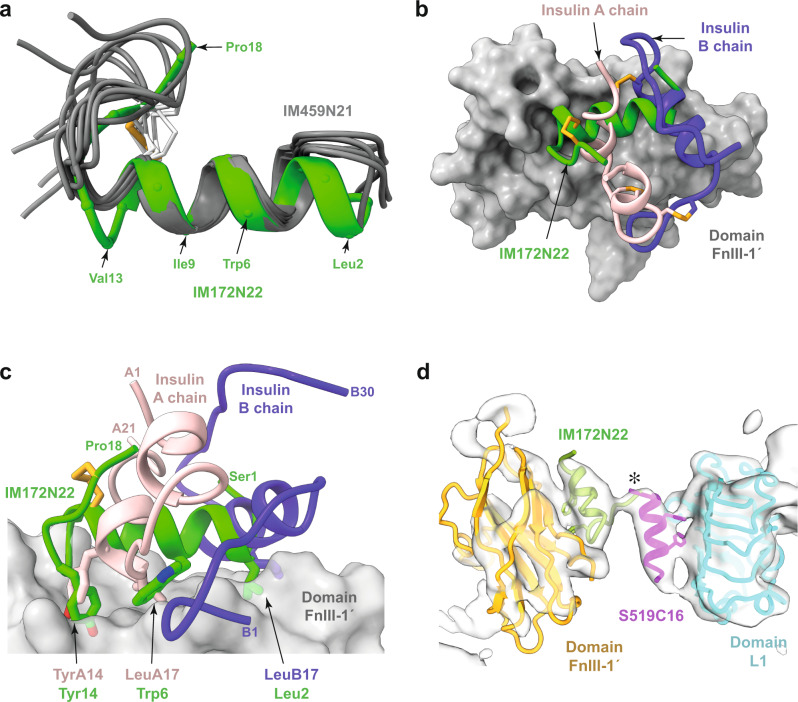
IM459N21 solution structure and comparison of the modes of binding of IM172N22 and insulin. **a** Correspondence of the backbone conformation of the hIR-bound IM172N22 peptide (green) with that of IM459N21 peptide (grey) determined in solution (for clarity, only six of the NMR-based IM459N21 models are shown). The orange bonds depict the IM172N22 cysteine residues and their associated disulfide bond; the white bonds depict those of IM459N21. Selected IM172N22 residues are labelled. **b** Overlay of the structure of IM172N22 (green) bound to domain FnIII-1′ (grey) with that of insulin (A chain pink, B chain dark blue) bound to the same domain (PDB 6SOF)^5^. The orange bonds depict the disulfide linkages within IM172N22 and within insulin. **c**, Correspondence of key [FnIII-1′]-engaging residues of IM172N22 with those of insulin. Chain termini are labelled and colors are as in panel **b**. **d** Proximity (asterisked) of the N terminus of a Site 1 peptide with that of the C terminus of a Site 2 peptide within the map of the IM459-bound hIR ectodomain. Peptides models are derived respectively from PDB 5J3H (S519C16 bound to the hIR L1-CR module^12^) and from the IM172N22-bound IRΔβ-zip structure presented in this manuscript. The IM172N22 peptide is in green, the S519C16 peptide is in light magenta, receptor domain colors are as in the primary structure domain layout provided in Supplementary Fig. 2a.

To test whether Site 2 peptides are intrinsically helical in solution, we used NMR to determine the solution structure of IM459N21 (see Methods). IM459N21 is seen to be α-helical across residues Glu3 to Glu12 and to have a conformation closely similar to that observed for IM172N22 in complex with receptor (Fig. 4a), suggesting that helicity upstream of the canonical disulfide bond of Site 2 peptides is likely a shared solution property.

IM172N22 engages the same FnIII-1 surface as that observed for the Site 2 insulin in the four-insulin-bound receptor structure^4,5^. However, there is no relationship between their manner of binding (Fig. 4b): the IM172N22 helix is oriented approximately orthogonal to those of insulin. However, there are some common elements: insulin TyrA14 docks into the same pocket as IM172N22 Tyr14, insulin LeuA13 engages the same pocket as IM172N22 Trp6, and insulin LeuB17 engages approximately the same surface as IM172N22 Leu2 (Fig. 4c). Nevertheless, beyond these, there appear to be no insulin equivalents to the remaining key residues of the D8 motif (Fig. 3c).

Finally, determination of the mode of binding of IM172N22 permits superposition of the peptide-bound FnIII-1′ domain onto the model of the IM459 complex—such superposition confirms not only correspondence of the Site 2 binding locations on domain FnIII-1′ but also the feasibility of polypeptide connection between the model Site 1 and Site 2 peptides (IM172N22 and S519C16, respectively; Fig. 4d).

### Site-specific mutagenesis of IM172N22

To test the above model further, we conducted alanine-scanning mutagenesis of IM172N22 at all sites except Ala7, Cys11, and Cys17 (Fig. 3d, Supplementary Fig. 7). Individual alanine substitution of Leu2, Trp6, and Tyr14 results in no detectable binding of the mutant peptide to hIR-A, of Glu3, Glu5, Ile9, Glu12, Val13 and Arg16 in a *ca* 10- to 100-fold reduction in *k*_i_ for hIR-A, and of Ser1, Glu4, Glu8, Glu10, Pro18, Pro19, Ser20, Glu21 and Ser22 in a lower than 5-fold reduction in *k*_i_. These data align qualitatively with the structural motif described above (Fig. 3b, c). Individual side-chain charge-reversal substitutions Glu3Arg, Glu5Arg, Glu12Arg, and Arg16Glu result in a similar or greater reduction in *k*_i_ than alanine substitution at these sites, aligning with the involvement of these residues in salt bridge interactions with domain FnIII-1. The Gly15Ala mutation results in a 10-fold reduction in *k*_i_. The changes in affinity resulting from these mutations broadly correlate with a corresponding reduction in ability to inhibit receptor phosphorylation by insulin (Fig. 3d).

We used far-UV circular dichroism (CD) measurement to determine whether the effect of any of the above mutations may have resulted from destabilization of the structure of the peptide itself (Supplementary Fig. 8). Five of the mutant peptides (Glu3Ala, Glu4Arg, Tyr14Ala, Arg16Ala, and Arg16Glu) were judged to have reduced helical content compared to the control peptide and hence part of their reduced *k*_i_ may be attributable to increased entropy of the mutant free peptide.

### Molecular dynamics

We also performed molecular dynamics simulations of IM172N22 in complex with an isolated domain FnIII-1 (see Methods for full details). In each of the ten independent 1000 ns simulations, the interaction of IM172N22 with domain FnIII-1 was stable and its helical secondary structure remained intact (Supplementary Fig. 9). The salt bridges described above remained mostly intact throughout the simulation period. The MD simulation thus supports the cryoEM model and indicates a well-formed and specific interaction of the peptide with the receptor.

## Discussion

The modes of Site 1 and Site 2 peptides interaction with hIR are distinct from those of insulin: Site 1 peptides displace a receptor element (αCT) from the receptor domain L1 surface whereas an exemplar Site 2 peptide IM172N22 is shown here to bind in a non-insulin-like fashion to insulin’s initial (and likely transient^14,25^) binding site on the receptor. Our exemplar [Site 2]-[Site 1] fusion peptide IM459 (a potent receptor agonist) is then seen to cross-link these receptor elements and in the process release receptor domains L1 and L1′ from their respective adjacent domains FnIII-2′ and FnIII-2. Release of these interactions is central to the signalling event^19^. The ultimate location of the αCT elements displaced by the respective [Site 1] components of IM459 is not resolved in our map—these elements, although cross-linked by the inter α-chain disulfide(s) at hIR residues Cys682-Cys683-Cys685^26^, lie downstream of largely unstructured regions of the respective α-chain insert domains and are indeed unlikely to be ordered within the IM459-bound receptor ectodomain (Fig. 2b).

The effect of the Site 1 and Site 2 peptides and their [Site 2]-[Site 1] fusions on insulin binding is illuminating. Unlike insulin itself, which at low concentrations increases the rate of dissociation and at higher concentrations decreases it^16^, the fusion peptides appear to stabilise pre-bound insulin across the concentration range we explored (Supplementary Fig. 2d–g). This result is readily explicable if one considers a single insulin molecule bound to one side of the receptor and the fusion peptide locking this conformation upon binding the other receptor side. Interestingly, a site 2 peptide alone also stabilizes pre-bound insulin (Supplementary Fig. 2g), whereas a site 1 peptide is not able to do so (Supplementary Fig. 2h), suggesting that the stabilization of the pre-bound insulin is driven by the binding of the site 2 portion of the fusion peptide. In fact, our cryoEM structure of the co-complex of hIR with both IM172N22 (a site 2 peptide) and insulin provides an exemplar of such stabilization.

The reconstruction of the IM459-bound receptor ectodomain shows very little density arising from domains FnIII-2 and FnIII-3 and their counterparts on the opposing monomer, implying a degree of mobility of these domains within this complex. This contrasts with the cryoEM structures of the insulin-bound receptor ectodomain, all of which display some ordering of these membrane-proximal elements^25^. Indeed, liganded structures have been obtained that place domains FnIII-3 and −3′ in close proximity at the point of membrane entry (*ca* 15 Å apart)^3^, though other structures, such as those with four bound insulins, display the domains further apart at the point of membrane entry (*ca* 35 Å apart)^4^. It is thus possible that [Site 2]-[Site 1]-peptides such as IM459 and S519 are unable to stabilize the receptor ectodomain fully in its activated form despite their relatively high affinity. Such reduced signalling output aligns with the EC_50_s reported in Fig. 1c. The structural reasons for [Site 2]-[Site 1]-peptides not being able to induce a fully activated (“legs-together”) conformation most likely result from the constituent domains of the receptor [L2-(FnIII-1)]_2_ head module being cross-linked differently by these peptides (Fig. 2g) in comparison to that by insulin (Fig. 2e). hIR thus appears to be molecule capable of being stabilised in a variety of conformations that in turn determine signalling efficiency.

Intriguingly, our structures also provide insight into how [Site 1]-[Site 2] fusion peptides might function as antagonists: molecular modelling reveals that such a peptide could lock the receptor into an apo conformation (Supplementary Fig. 4f).

The Site 1 peptide receptor-binding motif comprises two pairs of aromatic residues surrounding a single (non-specific) residue (Fig. 1a), all embedded within a helical peptide that engages the central β-sheet surface of hIR domain L1. The Site 2 motif is more complex—involving not only an α-helical element but also a C-terminal disulfide-linked loop element that directs the side chains of Val13 and Tyr14 into a pocket formed between the two β sheets of domain FnIII-1′. However, both these motifs may be mimicable by small-molecule helical mimetics, and the intrinsic inter-domain flexibility of hIR suggests that there may be a number of ways of chemically cross-linking them to achieve the desired signalling output of an insulin mimetic.

## Methods

### Peptides

Peptides were synthesized under contract by CPC Scientific, except for the N-terminally acetylated version of IM172N22 and the Glu3Arg, Glu3Ala, Glu4Arg, Glu4Ala, Glu5Ala, Glu5Arg, Trp6Ala, Gln8Ala, Ile9Ala, Glu10Ala, Glu10Arg and Tyr14Ala mutants of IM172N22, which were synthesized by Mimotopes (Australia). All syntheses employed standard solid-phase synthetic methods and peptides were isolated as lyophilized powders. LC-MS analyses demonstrated purities greater than 95% and M/z values consistent with that predicted for the desired sequence (see Source Data provided with this manuscript). BHI (Humalin®; Eli Lilly) was used as formulated (in 2.5 mg mL^−1^ *m*-cresol, 16 mg mL^−1^ glycerin, 0.015 mg/100 units zinc, sodium hydroxide and/or hydrochloric acid added to adjust the pH to the range 7.0-7.8).

### holoIR-A: Production and purification

Cellular membranes were prepared from human embryonic kidney (HEK293; ATCC #CRL-1573) cells stably transfected with cDNA corresponding to human IR-A (holoIR-A) plus a C-terminal C9 tag (TETSQVAPA) inserted within a pcDNA3.1 plasmid (Invitrogen), with positive clones selected with 800 μg mL^−1^ neomycin and confirmed by Western Blot. Frozen cell pellets were thawed in ice-cold homogenization/resuspension buffer (50 mM Tris-HCl, pH 7.5) containing one cOmplete® protease inhibitor tablet with EDTA (Roche Diagnostics) per 50 mL buffer. Cells were homogenized with an overhead motor-driven Teflon-glass homogenizer using 15–20 strokes, followed by centrifugation at 1100 × *g* for 10 min at 4 °C. The supernatant was saved on ice, pellets were homogenized as before, and centrifuged at 1100 × g for 10 min at 4 °C. Both supernatants were combined and subsequently centrifuged at 35,000 × *g* for 60 min at 4 °C. The pellet was resuspended in buffer (4 to 5 mL g^−1^ of starting cell paste) containing protease inhibitors and quick frozen in liquid nitrogen prior to storage at −80 °C. Protein concentration was determined using a BCA kit (Thermo Scientific) with bovine serum albumin (BSA) as standard.

### holoIR-A: Binding assays

Receptor binding affinities (*K*_i_) were determined using a competitive radioligand binding assay with human recombinant (3-[^125^I]-iodotyrosyl-A14)-insulin (Perkin Elmer; 2200 Ci mmol^−1^). The assays were performed with a scintillation proximity assay (SPA) method using polyvinyltoluene (PVT) wheat germ agglutinin-coupled SPA beads (Perkin Elmer). Assay buffer containing 50 mM Tris-HCl (pH 7.5), 150 mM NaCl, 0.1% w/v fatty-acid free BSA was used for all compound testing and reagent preparation. Ten-point concentration response curves with three-fold serial dilutions of test samples or controls were prepared in the assay buffer. 25 μL of compound dilution were added to a 96-well, white, clear-bottom microplate (Corning) followed by addition of 50 µL radioligand (35-46 pM final assay concentration), 75 µL IR-A-containing membrane preparation (as described above; 0.15 μg/well), and 50 µL SPA beads (0.15 mg/well). Plates were sealed and shaken for 1 min on a plate shaker. Following a 10-h incubation and bead settling period at room temperature, radioactivity was determined using a MicroBeta Trilux scintillation counter (Perkin Elmer) and expressed as counts per minute (CPM). Unlabeled biosynthetic human insulin (BHI) was included on each plate of each experiment as a control. Samples were tested in three independent assay runs on three different days.

The median maximum binding response (MAX) was determined in 8 wells/plate using assay buffer only and the median minimum binding or nonspecific response (MIN) was determined in 8 wells/plate using 100 nM BHI. All test sample concentration responses were normalized to the control response and calculated as a percent of the maximal response after correcting for nonspecific binding as shown below:

$$\%{{{{{\rm{Specific\; Inhibition}}}}}}=100\%-[({{{{{\rm{CPM}}}}}}-{{{{{\rm{MIN}}}}}})/({{{{{\rm{MAX}}}}}}-{{{{{\rm{MIN}}}}}}){{{{{\rm{\times }}}}}}100\%]$$

The concentration causing 50% inhibition of binding (IC_50_) was determined from four-parameter logistic non-linear regression analysis using the Analyzer utility within the Genedata Screener software package (version 17.0.2). The affinity constant (*K*_i_) was calculated from the IC_50_ value using the equation:

$${K}_{{{{{{\rm{i}}}}}}}={{{{{{\rm{IC}}}}}}}_{50}/(1+{{{{{\rm{L}}}}}}/{K}_{{{{{{\rm{d}}}}}}})$$

where L was the radioligand concentration used in the experiment determined for each experiment by counting aliquots of the radioligand mix and *K*_d_ the equilibrium binding affinity constant of the radioligand determined from saturation binding analysis (hIR-A *K*_d_ = 0.218 nM).

Percent specific binding (y-axis) was plotted vs log concentration of compound (x-axis) using Prism Version 9.3.1 (GraphPad Software). Individual measurements are provided as Source Data.

### holoIR-A: Auto-phosphorylation assays

Phosphorylation was quantified using an ELISA technique. Ninety-six well, flat-bottom microplates (Greiner Bio-One) were pre-coated with 2 µg mL^−1^ anti-C9 monoclonal antibody (Rho 1D4; University of British Columbia)^27^ in 20 mM sodium carbonate, pH 9.6, at 4 °C for overnight prior to the assay. On the assay day, plates were washed 3× with 0.1% Tween in 50 mM Tris Buffer Saline, pH 7.5 (TBST), and then blocked for greater than 1 h at room temperature (RT) with 1% BSA (Sigma) in TBST.

Stably transfected HEK293 cells over-expressing human holoIR-A containing a C-terminal C9 epitope tag (TETSQVAPA), were plated at 6 × 10^4^ cells per well serum-starved using 0.1% BSA in DMEM (Gibco) for overnight at standard tissue culture condition. On the assay day, cells were treated with 3-fold serially diluted assay control (BHI) and test compounds at 37 °C for 1 h, followed by quick rinse with ice-cold PBS, then lysed with 1% NP40 in 50 mM Tris, pH 7.4, 150 mM NaCl containing both Roche Complete™ Protease Inhibitors and 2 mM sodium vanadate. After a 15-min incubation in lysis buffer at 4 °C, the lysate was transferred to anti-C9 antibody coated capture plate (Rho 1D4; University of British Columbia)^27^. Following a 1 h incubation on the capture plates at RT, the unbound lysates were removed by rinsing 3× with TBST followed by addition of horseradish peroxidase-conjugated anti-phosphotyrosine-secondary antibody 4G10-HRP™ (1:5000 dilution; Millipore catalog #16-105, lot #3316045) in TBST containing Roche Complete™ protease inhibitors and 40 µM vanadate. After a 1 h incubation at room temperature, the secondary antibody was removed by washing 3× with TBST. Signal detection occurred with the addition of TMB, an HRP substrate (Pierce). After 5–10 min, the reaction was stopped by adding an equal volume of 2 M H_2_SO_4_, and the absorbance readings at 450 nm (A_450_) were recorded using a Envision plate reader (PerkinElmer). Samples were tested in three independent assay runs on three different days.

Assays were performed with a single replicate 11-point concentration-response curve (CRC) per experiment for the test samples with a single 11-point CRC for each positive control (BHI) on each plate. The MIN response was determined in 4 wells/plate and the MAX response was determined in 4 wells/plate using a maximally efficacious concentration of BHI (100 nM). A_450_ values for the concentration response curves were converted to percent relative to the 100 nM BHI response by subtracting the median MIN A_450_ from the test sample A_450_ values and dividing by the median A_450_ with 100 nM of BHI (MAX minus MIN) as shown below:

$$\%{{{{{\rm{Specific\; Response}}}}}}=({{{{{\rm{Absorbance}}}}}}-{{{{{\rm{MIN}}}}}})/({{{{{\rm{MAX}}}}}}-{{{{{\rm{MIN}}}}}}){{{{{\rm{\times }}}}}}100\%$$

Concentration resulting in 50% of the maximal response (EC_50_) values in the presence of BHI were determined from 4-parameter logistic non-linear regression analysis using the Analyzer utility within the Genedata Screener software package (version 17.0.2).

Inhibition of phosphorylation of hIR by the peptides studied was assay using an experimental design equivalent to that above, except that the receptor was co-incubated with BHI at a concentration equivalent to its EC50.

Percent specific response (y-axis) was plotted versus the log concentration of compound (x-axis) using Prism Version 9.3.1 (GraphPad Software). Individual measurements are provided as Source Data.

### holoIR-A: Accelerated dissociation assays

Following a procedure adapted from De Meyts^16^, [holoIR-A]-expressing IM-9 human lymphoblast cells (ATCC® CCL-159™) were cultured at 37 °C in 5% (v/v) CO_2_ humidified atmosphere in RPMI 1640 medium without L-glutamine (Thermo Fisher) supplemented with 10% (v/v) fetal bovine serum (FBS), 2 mM L-glutamine (Hyclone), 1× antibiotic / antimyotic solution (Hyclone). IM-9 cells (5 × 10^7^ cells/mL) were centrifuged at 14.7 x *g* for 5 min at 4 °C and resuspended in 500 µl HBB buffer comprising 100 mM HEPES, 120 mM NaCl, 5 mM KCl, 1.2 mM MgSO_4_, 1 mM EDTA, 10 mM glucose, 15 mM NaOAc and 1% (v/v) BSA, pH 7.6. Ice-cold 15-25 pM ^125^I-insulin solution was added to IM-9 cells and incubated at 4 °C for 2.5 h for pre-bound experiments. Biosynthetic human insulin (BHI) and compounds were serial diluted in HBB buffer starting at 30 µM with 1:3 dilution factor. All diluted BHI control and compounds were equilibrated at 16 °C. Following receptor and ^125^I-insulin pre-bound, the mix was quickly centrifugated and supernatant was removed. The bound ^125^I-insulin and IM-9 cells were then resuspended with 16 °C pre-equilibrated HBB, pH 7.6. The ^125^I-ligand/IM-9 cell mixture was aliquoted to cold compounds at 1:40 ratio, respectively. The reactions were incubated at 16 °C for 30 min to allow dissociation of prebound ^125^I-insulin and then harvested by centrifugation and aspiration of the supernatant. The radioactivity of cell pellets was determined by gamma counter (Perkin Elmer). Samples were tested in three independent assay runs on three different days. Individual measurements are provided as Source Data.

### IR-A^ecto^: Protein production and purification

IM459 was complexed with an IR-A ectodomain construct (“IR-A^ecto^”) spanning residues 1-914 of the receptor, with IR-A^ecto^ being produced from the same CHO Lec8 stable cell line as described previously^28^, these cells being obtained from CSIRO (Australia) by M.C.L.’s institution (WEHI) under a Technology Transfer Agreement. Cells were thawed and then passaged four times before expanding into ten T150 tissue culture flasks, employing as medium DMEM (High glucose, no glutamine; Lonza) plus 10% dialysed fetal bovine serum (FBS; Life Technologies), 25 μM methionine sulphoximine and 1x GS supplements (Merck). When the cells were 90% confluent, they were then trypsinized and half the cells of each respective flask used to inoculate respectively a total of twenty 850 cm^2^ roller bottles. The roller-bottle culture supernatant was then harvested on day 20, centrifuged 6238 x *g* for 20 min, and then decanted and filtered through Stericap bottle top filters (Merck Millipore), with PMSF (1:1000 dilution of 100 mM PMSF/propan-2-ol; Merck), and sodium azide (Sigma-Aldrich) to 0.02% being added prior to volume reduction. Sample volume reduction was achieved by cycling the conditioned medium at room temperature through a stack of two Pellicon 3, 0.11 m^2^, 10 kDa concentrator cartridges (Merck Millipore) until the concentrate volume was 500 ml.

The IR-A^ecto^ complex was then purified from the concentrated, conditioned cell-culture media by elution with IM459 from an insulin-affinity column^3^, followed by size-exclusion chromatography. In particular, the Bis-boc-insulin Mini-Leak beads (∼2 mL column) were reconstituted in TBSA, washed with 1.5 bed volumes (“BV”) 6 M guanidine HCl followed by 10 BV TBSA and then equilibrated in TBSA in preparation for IR-A^ecto^ purification. Ten-fold-concentrated culture supernatant (20 mL) was then passed through the column twice and washed with ∼25 BV TBSA. The column was then washed with 3 BV of a solution comprising 0.2 M citrate, 0.4 M NaCl (pH 5.0) plus 0.02% sodium azide, chased with 3 BV TBSA, and collected into 3 M Tris (1.8 mL). Remaining (high-affinity) IR-A^ecto^ material was then eluted with IM459 solution (60 μM in TBSA, 2.5 BV), chased with diluted IM459 solution (12 μM in TBSA, 2.5 BV), and then with TBSA (2.5 BV), collected into 3 M Tris (1.8 mL). The column was then cleaned with 3 BV of a solution comprising 0.3 M citrate (pH 3.0) plus 0.02% sodium azide and collected into 3 M Tris solution (1.8 mL). The crude eluants were concentrated to ∼500 μL and visualised by SDS-PAGE (10 μL aliquots). The IM459 elutions were collected, concentrated to 200 μL, and purified by isocratic size-exclusion chromatography (SEC) in TBSA. IM459 was retained through chromatography as visualised by SDS-PAGE (Fig. 2a; see Source Data for uncropped gel image) and fractions containing IR-A^ecto^ and IM459 were combined and concentrated. The complex was then concentrated to 0.1 mg mL^−1^ in preparation for cryoEM imaging.

### Cloning and production of IRΔβ-zip

The cloning and production of the leucine-zippered IR-A ectodomain construct “IRΔβ-zip” has been described previously^3^ and the material used here was from the same batch; nevertheless, the protocols are reproduced here for ease of reference. A gene encoding IRΔβ-zip (residues 1-916 of the insulin receptor IRΔβ construct^20^ followed at its C terminus by the 33-residue GCN4 zipper sequence RMKQLEDKVEELLSKNYHLENEVARLKKLVGER^22^ and inclusive here of the population variants Tyr144His, Ile421Thr, and Gln 465Lys^29,30^) was synthesized by ATUM (USA) and then cloned into the pEE14 mammalian expression vector (Lonza) for stable expression of the protein in CHO Lec8 cells. Cells were transfected with complexes of plasmid DNA and X-tremeGENE 9 transfection agent (Roche) and then selected with 25 μM methionine sulphoximine in Lonza DMEM (High Glucose) medium containing 1 × GS supplement (Merck; *i.e*., “single strength”) and 10% dialysed fetal bovine serum (FBS; Life Technologies). Cells were plated in 96-well plates using limiting dilution and colonies allowed to form over several weeks. Secretion of target protein from colonies was detected via Western Blots using anti-hIR mAb 83-7 at a concentration of 0.1 μg mL^−1^ (hybridomas expressing mAb 83-7^21^ were a gift from Professor Ken Siddle, Cambridge, UK; the epitope of mAb 83-7 lies within the cysteine-rich domain of the hIR ectodomain and the product from the hybridoma has been authenticated via crystallization of its Fab fragment in complex with IRΔβ^20^). Dozens of colonies were amplified in twelve-well trays and later in tissue culture flasks and monitored for expression of IRΔβ-zip. Several of the best-expressing clones were then screened further by seeding cells at exactly the same densities in six-well trays and individually monitored for expression over time. The single best-expressing clone was then selected to enter roller bottle scale-up. For scale-up, a richer medium containing 15% dialysed FBS and 2× GS supplement (*i.e*., “double strength”) was used. Cells were seeded in roller bottles and allowed to grow for 14 days. 2.5 mM valproic acid (Sigma) was added at this point and the cells were allowed to incubate for a further 7 days, at which stage the conditioned medium was decanted from the roller bottles and then filtered for purification.

Purification of IRΔβ-zip was by affinity chromatography and SEC as described above for the IM459-IR-A^ecto^ complex, but with eluting from the insulin-affinity column with human insulin (at 20 μM concentration and then 4 μM concentration; Sigma-Aldrich, catalog no. 91077 C)^3^ rather than with IM459.

### Cloning and production of Fv 83-7

The cloning, production, and purification of Fv 83-7 has been described previously^3,12^ and the material used here was from the same produced and purified batch; nevertheless, the protocols are reproduced here for ease of reference. Fv 83-7 was prepared using a *Brevibacillus* expression system. Briefly, codon- and expression-optimised DNA corresponding to murine monoclonal antibody 83-7 variable heavy (VH) chain residues 1-118^20,21,31^ followed by the sequence SLVPRGSSSEQKLISEEDLN (thrombin cleavage site + c-myc tag) was synthesized and then cloned into the vector pCDNA3.1 by DNA2.0 (USA). Similarly, DNA encoding the 83-7 variable light (VL) chain residues 1-112^20,21,31^ followed by the sequence SSDYKD (FLAG tag) was synthesized and then cloned into the vector pJ201 (DNA2.0, USA). Both genes were then individually transferred into the BamH1/Xba1 sites (in frame with a secretion signal) of the plasmid pNCM02 (Takara Bio, Japan) for independent transformation into *Brevibacillus choshinensis* cells (Takara Bio, Japan). Isolated colonies of the transformed *B. choshinensis* cells were then screened for over-expression of the expected domain by Western blot using antibodies 9E10 and KM5-1C7-8-5 (respectively) at a concentration of 0.1 μg mL^−1^—antibody KM5-1C7-8-5 is an in-house anti-FLAG antibody (WEHI Antibody Facility; Australia), antibody 9E10 is an anti-c-myc tag monoclonal antibody^32^ (ATCC CRL-1729). The highest-expressing colonies were then stored as glycerol stocks. For 1 L scale-up, each glycerol stock was used to inoculate 2 mL of 2SY broth containing 10 μg mL^−1^ neomycin sulfate (Sigma Aldrich, USA) (2SYnm), followed by incubation overnight at 30 °C at 120 rpm on a rotating platform. 0.2 mL of these respective cultures were then used to inoculate a further 20 mL of 2SYnm broth and, once sufficiently grown, 5 mL of this inoculum was used to inoculate a further 500 mL of 2SYnm broth in Tunair™ flasks (Sigma Aldrich, USA). Cultures were incubated for 72–96 h, with 1 mL samples taken at 24 h intervals to monitor production via SDS-PAGE and Western blot. Optical density was monitored at 660 nm. Samples were centrifuged at 16.2 x *g* for 5 min to pellet bacteria and to recover the supernatant containing the secreted product. 700 mL of conditioned medium containing the c-myc-tagged 83-7 VH domain was then combined with 850 mL of conditioned medium containing FLAG-tagged 83-7 VL domain and incubated for 30 min at room temperature, followed by addition of 3 M Tris HCl (pH 8.5) at the ratio of 5 ml L^−1^ of combined media. This process was estimated to give a slight excess of VL monomers in the VL/VH mixture. The pH-adjusted combined media were then run through an in-house 9E10-conjugated Mini-Leak Low affinity column (Kem En Tec, Denmark)^33^ and the desired Fv 83-7 eluted with c-myc peptide (decameric form) prepared in Tris-buffered saline plus azide (24.8 mM Tris-HCl pH 8.0, 137 mM NaCl, 2.7 mM KCl plus 0.02% NaN_3_; TBSA). Fractions were combined with one tablet of cOmplete protease inhibitor cocktail (Roche, Switzerland). Fractions from Superdex 200 10/300 SEC (GE Healthcare Life Sciences) examined by SDS-PAGE showed the presence of two bands of molecular weight 14 and 16 kDa, respectively, indicating a correctly-formed Fv eluting at 22 kDa. The c-myc tag was then removed from the 83-7 VH domain of the Fv 83-7 as follows. The Fv 83-7 was diluted to ~ 4 mg mL^−1^ in TBSA and then combined with 10 mM CaCl_2_ and 0.5 U human thrombin per mg Fv 83-7 (Roche, Switzerland). The sample was incubated at 37 °C for 4 h and the reaction stopped by the addition of 1 mM phenylmethylsulfonyl fluoride and by incubation on ice. The sample was then re-purified by means of a Superdex S75 column (GE Healthcare Life Sciences, USA).

### IRΔβzip + insulin + Fv 83-7 + IM172N22 complex formation

Three mol equivalents of Fv 83-7 and five mol equivalents of insulin were added to the insulin-eluted fraction of IRΔβ-zip and the complex incubated at 4 °C for 30 min. The complex was then concentrated to 200 µL and purified by SEC using a Superdex 200 Increase column (Cytiva) with isocratic TBSA elution. Pure fractions containing IRΔβ-zip (dimeric form), insulin and Fv 83-7 (as assessed by SDS page) were combined and then mixed at a 1:1 volumetric ratio with IM172N22 prepared at 4 mg mL^−1^ in 10 mM HCl, providing a vast molar excess of the peptide to the solution.

### CryoEM grid preparation: IM459 and IM172N22 receptor complexes

4-μL volumes of the respective samples were applied to UltrAuFoil R1.2/1.3 300-mesh grids (Quantifoil Micro Tools GmbH; Germany) that were prior glow discharged in a Pelco easiGlow device (Ted Pella; CA) at 10 mA for 90 s (IM172N22 complex) or at 15 mA for 30 s (IM459 complex). Grids were blotted for 3 s at −3 blot-force setting in a Vitrobot mark IV (ThermoFisher Scientific; operated at 4 °C and 100% humidity) before being plunge frozen in liquid ethane.

### CryoEM dataset collection: IM459 complex

CryoEM imaging was performed using a Titan Krios (ThermoFisher Scientific) equipped with a Gatan K2 Summit™ camera, a Quantum-GIF energy filter and the EPU 2 data acquisition software. Imaging was performed in nanoprobe energy-filtered zero-loss mode using a 20 eV slit width. A nominal magnification of 130,000× was used which provided a calibrated specimen level pixel size of 1.06 Å. A C2 condenser aperture of 50 μm was used and the K2 camera was operated in counting mode at a dose at a rate of 8 e^−^.pixel^−1^ s^−1^. The total exposure time of 10 s was fractionated into 50 sub-frames, resulting in a total accumulated dose of 69.5 e^−^ Å^-2^ per movie. Movies were collected at defocus ranges of −0.5 μm to −1.5 μm and −2.0 μm to −4 μm using the beam-image shift method, with nine movies collected per stage shift. All movies were collected from a single grid (*n* = 1).

### CryoEM 3D reconstruction: IM459 complex

The dataset of 5848 movies obtained from the IM459-complexed receptor was processed entirely within cryoSPARC version 2^34^. Movies were patch motion-corrected and contrast transfer function (CTF) parameters were then estimated using the “patch-CTF” tool. Particles were picked from 126 high-defocus micrographs (>3 Å under-focus) using the “blob-picker” tool and then subjected to two-dimensional (2D) classification to provide templates for “auto-picking” the entire set of micrographs. 2.5 M particles were selected, extracted with 5× binning and classified into 200 2D classes. 495 k particles from “good” classes were retained and subjected to ab initio 3D reconstruction with four classes. Two 3D classes were used to create templates for a second round of template-based auto-picking, which then extracted 2 M particles at full resolution and which were then classified into 200 2D classes. 552 k particles from 18 “good” classes were retained and subjected to ab initio reconstruction into four 3D classes. One 3D class containing 176 k particles was then progressed into refinement. A round of heterogeneous refinement with two decoy 3D classes (generated from “bad” particles) was used to clean this 3D class, retaining 126 k “good” particles. A round of heterogeneous refinement using particles from the remaining three ab initio classes was then conducted, recovering a further 70 k particles for the best class. The combined particles were subjected to 3D refinement and local refinement to give a nominally 5.44 Å reconstruction. These particles were re-extracted at 400 px box size and refined to improve marginally to 5.40 Å with 184 k particles. A softened mask was generated covering the binding site (the L1-CR-L2 module and domain FnIII-1′) and a further round of local refinement gave a nominally 5.03 Å resolution focused reconstruction. Further detail is provided in Supplementary Fig. 3 and Supplementary Table 1.

### NMR solution structure: IM459N21

All NMR spectra were recorded on a Bruker Avance III 600 MHz spectrometer equipped with a QCI-F CryoProbe (Bruker, Billerica, MA). Neat dry powder of the peptide (purchased from CPC Scientific, San Jose, California, USA) was dissolved in a buffer containing 20 mM Tris-d_11_, pH 7.4, 100 mM NaCl, 5% D_2_O to reach a final peptide concentration of 0.75 mM, before being transferred into a 5 mm NMR tube. The following 2D NMR experiments were then performed: (i) COSY (no. of transients = 16, *t*_2_ dimension = 4096, *F*_2_ spectral width = 12 ppm, *t*_1_ dimension = 1024, *F*_1_ spectral width = 12 ppm, recycling delay = 2 s), (ii) TOCSY (mixing time = 60 ms, no. of transients = 32, *t*_2_ dimension = 2048, *F*_2_ spectral width = 12 ppm, *t*_1_ dimension = 256, *F*_1_ spectral width = 12 ppm, recycling delay = 2.1 s), (iii) NOESY (mixing time = 300 ms, no. of transients = 128, *t*_2_ dimension = 2048, *F*_2_ spectral width = 12 ppm, *t*_1_ dimension = 256, *F*_1_ spectral width = 12 ppm, recycling delay = 2.1 s), (iv) NOESY (mixing time = 400 ms, no. of transients = 64, *t*_2_ dimension = 2048, *F*_2_ spectral width = 12 ppm, *t*_1_ dimension = 256, *F*_1_ spectral width = 12 ppm, recycling delay = 2.1 s), and (v) ^13^C-HSQC (no. of transients = 64, *t*_2_ dimension = 2048, *F*_2_ spectral width = 16 ppm, *t*_1_ dimension = 512, *F*_1_ spectral width = 125 ppm, recycling delay = 1.6 s). All experiments were performed at 298 K and NMR spectra were processed using NMRPipe^35^ version 10.9 revision. 2021.258.11.26. A solvent filter was applied in the direct dimension, followed by a Gaussian apodization function, zero-filling to double the number of acquired points and phase correction. The indirect dimension was processed with a cosine apodization function and zero-filling to double the number of acquired points. A polynomial baseline correction was subsequently applied to the direct dimension. The ^13^C-HSQC spectrum was analyzed and assigned using the CCPNMR suite (version 2)^36^. Spin systems were identified using a combination of NOESY and TOCSY spectra, where NOESY cross-peaks were used to assign inter-residue connections.

Structure calculations were performed using the GROMACS molecular dynamics suite (version 5.0.7)^37^ and the AMBER99SB force field^38^. Distance restraints from NOE cross-peaks for IM459N21 were generated and calibrated using the CCPNMR suite. Only NOE cross-peaks with unambiguous assignments were used for the structure calculations. NOE distance restraints were iterated until a converged structure ensemble was achieved with a backbone RMSD of 0.44 Å. Dihedral angle restraints were generated based on chemical shift data from backbone atoms using the DANGLE software within the CCPNMR suite (version 2; see Supplementary Fig. 10). Solvation of IM459N21 was done using 7179 water atoms with TIP3P topology. The system was rendered neutral by adding one chloride ion. The initial stapled peptide structure was prepared for structure calculations by energy minimization followed by short equilibration simulations at constant volume or pressure. For the non-natural amino acids at positions 0 and 7, the amino acids were generated as separate non-natural amino acids and parametrized for GROMACS using ACPYPE (beta version)^39^. After energy minimization, bond restraints were applied for the Sγ atoms of Cys11 and Cys17, and a short GROMACS simulation was run using these bond restraints, whereupon the covalent disulfide bond was generated using built-in functions in GROMACS. Structure calculations were then performed iteratively, where fifteen structures of the peptide were calculated using distance and dihedral angle restraints followed by evaluation using the CCPNMR suite, where restraints were reassigned or removed. Restraints with correct assignment and upper-bound violations were iteratively recalibrated by applying a soft upper-bound restraint. This was repeated until a converged structure ensemble was reached, of which sixteen structures were chosen as a representative ensemble based on Ramachandran backbone dihedral angles, distance restraint violations, and a converged backbone RMSD. Refinement statistics are provided in Supplementary Table 2.

### CryoEM initial atomic model: IM459 complex

Receptor domains L1 and CR and the S519C16 peptide were extracted from PDB entry 5J3H^12^ and were rigid-body docked into the map density using ChimeraX (version 1.2.5)^40^. Likewise, receptor domains L2, L2′, FnIII-1, and FnIII-1′ were extracted from PDB entry 6HN5^3^ and similarly rigid-body docked into the map density. The resultant molecular model was improved further once the structure of the IRΔβzip + insulin + Fv 83-7 + IM172N22 complex became available (see below).

### CryoEM dataset collection: IM172N22 complex

CryoEM imaging was performed using a Titan Krios (ThermoFisher Scientific) equipped with a Gatan K3 camera, a Quantum-GIF energy filter, and the EPU 2 data acquisition software. Imaging was performed in nanoprobe energy filtered zero loss mode using a 10 eV slit width. A nominal magnification of 130,000 × was used which provided a calibrated specimen level pixel size of 1.06 Å. A C2 condenser aperture of 50 μm was used and the K3 camera was operated in correlated double sampling mode at a dose rate of 10.6  e^−^.pixel^−1^ s^−1^. Datasets were collected using the beam-image shift method, with 21 movies collected per stage shift. Movies were collected using a 7 s exposure time fractionated into 60 sub-frames resulting in a total accumulated dose of 60 e^−^ Å^−2^ per movie. Movies were collected at a defocus range of −0.5 μm to −2.0 μm. All movies were collected from a single grid (*n* = 1).

### CryoEM 3D reconstruction: IM172N22 complex

A combined dataset of 7320 movies was motion-corrected using MotionCor2^41^ within RELION version 3.1^42^. CTF correction was completed using CTFFIND version 4.1.13^43^, also within RELION version 3.1. Images showing severe ice contamination or poor CTF fit were excluded, resulting in a final set of 6219 images. 2.9 M particles were picked using crYOLO^44^ and extracted in RELION version 3.1, binned 4x. The binned particles were exported for processing in cryoSPARC version 3^34^ wherein they subjected to 2D classification and heterogeneous refinement with two classes to remove bad particles. 855k particles were retained and converted to star format using csparc2star.py within the pyem suite^45^. All further steps were completed in RELION 4.0 (beta). The particles were re-extracted with a box size of 384 pixels and Fourier binned to 288 pixels, giving a pixel size of 1.413 Å.pixel^−1^. The particles were then refined using a consensus structure of the (peptide-free) IRΔβ-zip + insulin + Fv 83-7 complex^3^ as an initial model. A soft mask containing the domains L1, CR, L2, FnIII-1, human insulin, αCT´ L2´, and FnIII-1´ was generated from the refined map and used to 3D classify the particles into four classes with no alignment. One 3D class containing 346k particles was selected and then refined with the first reconstruction as an initial model. CTF refinement was then performed, with beam tilt, trefoil, and 4th order aberrations estimated first, followed by anisotropic magnification and finally per-particle defocus and per-micrograph astigmatism corrections. The particles were then refined again, followed by Bayesian polishing. Signal corresponding to domains FnIII-2, FnIII-3, L1′, CR’, FnIII-2′ and FnIII-3′ and to the domain CR’-associated Fv 83-7 module was judged heterogeneous and was removed through particle subtraction using RELION version 4.0 (beta)^46^. Density corresponding to the Fv 83-7 module bound to domain CR was similarly removed, to ensure that the reconstruction was not compromised by the mobility of the module. 3D refinement of the subtracted particles gave the final focused refinement with a resolution of 2.9 Å by gold-standard FSC. These steps are summarized in Supplementary Fig. 5.

### CryoEM atomic model: IM172N22 complex

Domains L1, CR, L2, FnIII-1, L2, and L2′ and the insulin moiety were extracted from PDB entry 6HN5^3^ and docked manually into the above map using ChimeraX (version 1.2.5)^40^, followed by flexible fitting with adaptive distance restraints in ISOLDE version 1.3.2^47^. Real-space refinement in PHENIX version 1.19.2^48^ followed, with PDB entry 6HN5 used to provide a source of secondary structure restraints. Using Coot, a single pose from the IM459N21 NMR ensemble was then docked into each of the discerned density features on the surface of domains FnIII-1 and FnIII-1′, respectively, followed by mutation of the sequence to that of IM172N22 sequence and by real-space refinement. The entire structure was then carefully adjusted using ISOLDE to fit density, followed by a final real-space refinement within PHENIX, in the presence of starting-model restraints, secondary-structure restraints, and Ramachandran restraints. Final refinement and reconstruction statistics are provided in Supplementary Table 1.

### Final molecular modelling: IM459 complex

The molecular model of the IM459 complex was improved further by incorporation of domains L2, FnIII-1, L2′ and FnIII-1′ and the IM172N22 peptide from the IM172N22 complex structure described above, these entities being docked rigidly into density using ChimeraX (version 1.3). The IM172N22 and the S519C16 chains were then mutated to the IM459 sequence using COOT version 0.9 and combined into a single polypeptide chain using the *Sphere Refine* and *Sphere Regularization* tools within COOT. These steps were then followed by a single round of real-space refinement within PHENIX in the presence of starting-model restraints, secondary-structure restraints, and Ramachandran restraints. Final refinement and reconstruction statistics are provided in Supplementary Table 1.

### Molecular dynamics (MD) simulations

Coordinates for IM172N22 in complex with IR domain FnIII-I (residues Asn470-Ala592) were extracted from the cryo-EM structure of the IM172N22 complex reported above, and used to carry out MD simulations in order to examine the structural integrity and peptide-receptor interactions. All computational works were performed using the modules in Schrödinger software suite (version 2020.3) with OPLS3e force field^49^. The protein preparation module was used to process the complex structure and to add any missing atoms. The N- and C-termini of domain FnIII-I were capped with acetyl and N-methyl amide groups, respectively. The protonation states of ionizable residues were determined at pH 7.0 using the PROPKA module. The assemblies were further energy minimized with restrained heavy atoms. The MD simulations were set up and run with the Desmond MD package within the Schrödinger suite. TIP3P waters with a buffer size of 10 Å were then added to the complex systems along with 0.15 M NaCl, the whole system containing 30 K atoms. The integration time was 4 fs with hydrogen mass repartition. The Desmond MD’s default seven-stage equilibration workflow was applied prior to the MD production runs. Ten MD runs of 1000 ns each at 300 K were performed for a total of 10 μs simulation time. For each MD run, 1000 snapshots were saved at 1 ns interval for trajectory analysis. All MD simulations were carried out with V100 GPU on the Amazon cloud.

### Far-UV CD spectroscopy: IM172N22 mutant peptides

Secondary structure characterization of Site 2 peptides in the presence of 10 mM phosphate buffer (pH 7.0) was undertaken using a J-1500 CD spectrometer (JASCO). Peptides of interest (0.2 mg ml^−1^) peptide were transferred into 0.2 cm path-length quartz cuvette for far UV-CD measurement at room temperature (25 °C). Spectra were collected in 250–190 nm wavelength range, and corrected by subtracting the appropriate control. [θ] ellipticity was converted to mean residual ellipticity using the formula [θ]_MRE_ = θ *MRW/cl, where MRW = (molecular weight of peptide)/(number of peptide bonds in the peptide). Secondary-structure quantitative analysis was undertaken using the built-in SSE multivariate analysis software. Spectra were recorded in standard “mdeg” mode with a scanning speed 50 nm min^−1^, a 2 s response and band width 2 nm. Individual measurements are provided as Source Data.

### Reporting summary

Further information on research design is available in the Nature Research Reporting Summary linked to this article.

## Supplementary information

Supplemetary Information

Reporting Summary

## Data Availability

The study made use of the following publicly available data sets: PDB entries 4ZXB, 5J3H, 6HN4, 6HN5, and 6SOF. Model coordinates and cryoEM maps generated in this study have deposited in the PDB and EMDB: IM459-complexed IR-A^ecto^: PDB code 7U6D, EMDB code EMD-26363; IM172N22-complexed IRΔβ.zip + Fv 83-7 + insulin: PDB code 7U6E, EMDB code EMD-26364; NMR structure of IM459N21: PDB code 8DI2. Source data are provided with this paper. Other raw data and biological materials will be made available subject to the negotiation of a suitable agreement setting forth the terms and conditions of transfer and use of any such materials.

## Source data

Source Data
