# Supplementary material for: Activation of the human insulin receptor by non-insulin-related peptides
Source: Nat Commun. 2022 Sep 28;13:5695. doi: 10.1038/s41467-022-33315-8 (PMC9519552; doi:10.1038/s41467-022-33315-8)
Supplement: Supplementary file 1 — Supplemetary Information [file 41467_2022_33315_MOESM1_ESM.pdf]

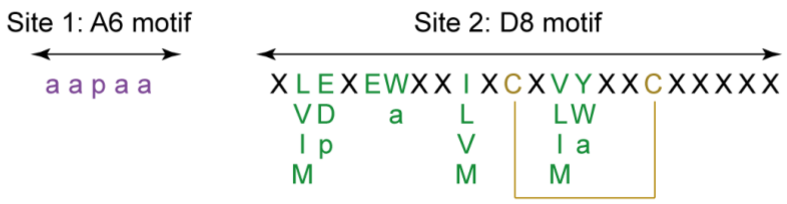

Supplementary Figure 1. **| Binding motifs of the Site 1 and Site 2 peptides.** The refined Site 1 and Site 1 peptide motifs are termed A6 and D8, respectively[1]. Defining residues are in *green* and *purple*, respectively, with the canonical disulfide-linked cysteines in *brown*. "a" represents an aromatic residue, "X" any residue, and "p" a polar residue.

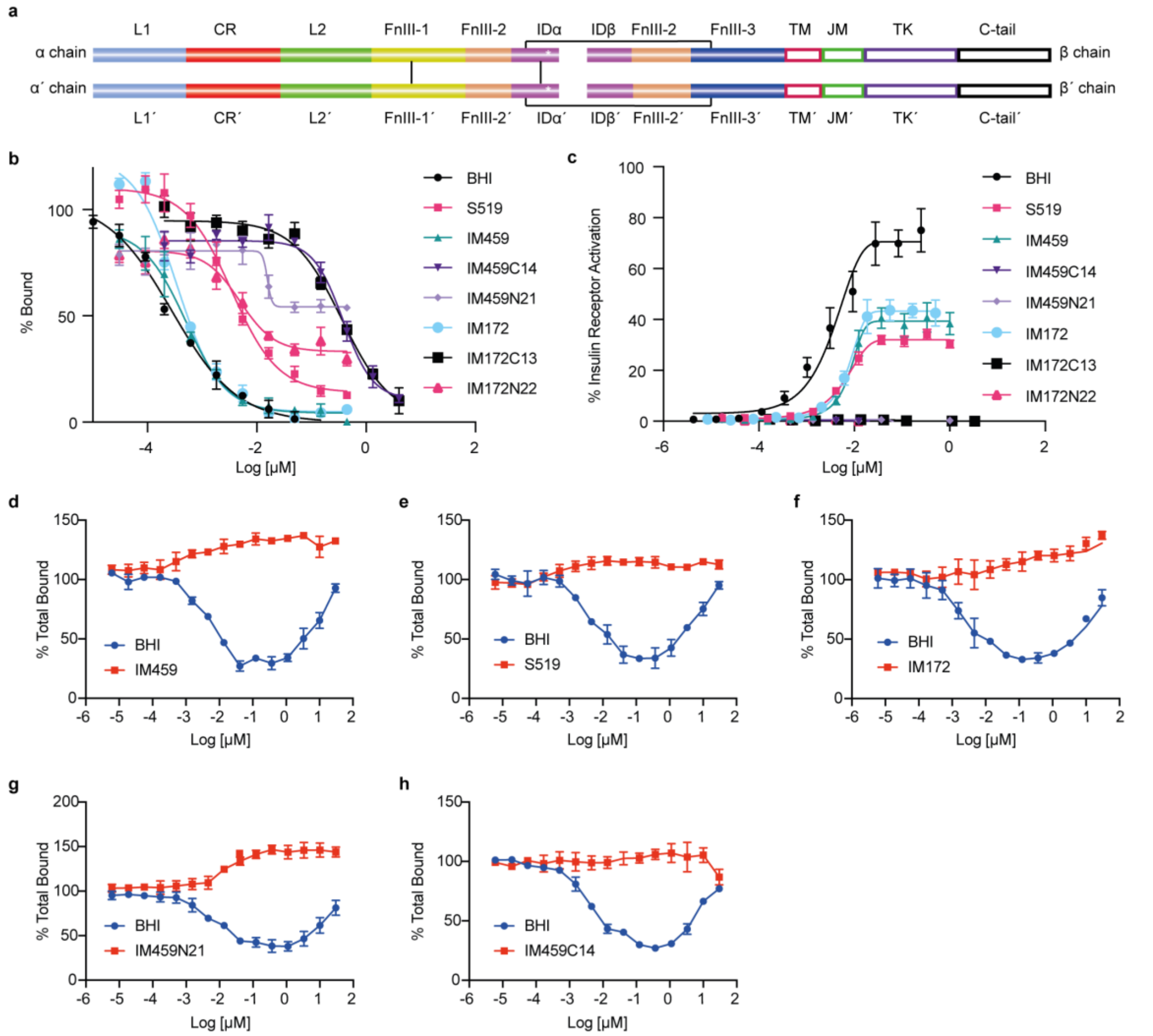

Supplementary Figure 2 | Domain structure of hIR and characterization of binding peptides. **a**, Receptor domains are L1, L2: first- and second leucine-rich-repeat domains; CR: cysteine - rich region, FnIII-1, -2, -3: first-, second- and third fibronectin type III domains, respectively; IDα, IDβ: α- and β-chain components of the insert domain; TM: transmembrane segment; JM: intracellular juxtamembrane segment, TK: tyrosine kinase domain; C-tail: C-terminal tail segment (domains in the second αβ monomer are suffixed with a prime symbol). Black connectors denote disulfide bonds. The C-terminal segments (αCT and αCT', respectively) of the α chains are indicated by white asterisks. In the construct IRΔβ-zip, the TM, JM, TK and C-tail domains are replaced by a leucine zipper element and the heavily O-glycosylated segment at near the N terminus of the β chain is deleted[2]. Panel is adapted and reprinted from Molecular Metabolism, v52, Michael C. Lawrence, "Understanding insulin and its receptor from their three-dimensional structures", p101255. Copyright Elsevier (2021), subject to http://creativecommons.org/licenses/by-nc-nd/4.0/. **b**, Peptide affinity determined using a competitive radioligand binding scintillation-proximity assay. **c**, Receptor activation determined using an ELISA assay. **d-h**, Dissociation of pre-bound radio-labelled insulin from surface-expressed hIR by peptides IM459, S519, IM172, IM459N21 and IM459C14, respectively. In all plots, error bars are standard errors of the mean, derived from $n = 3$ independent replicates. Where not visible, error bars are smaller than the markers. For full details, see Methods. Source data are provided as a Source Data file.

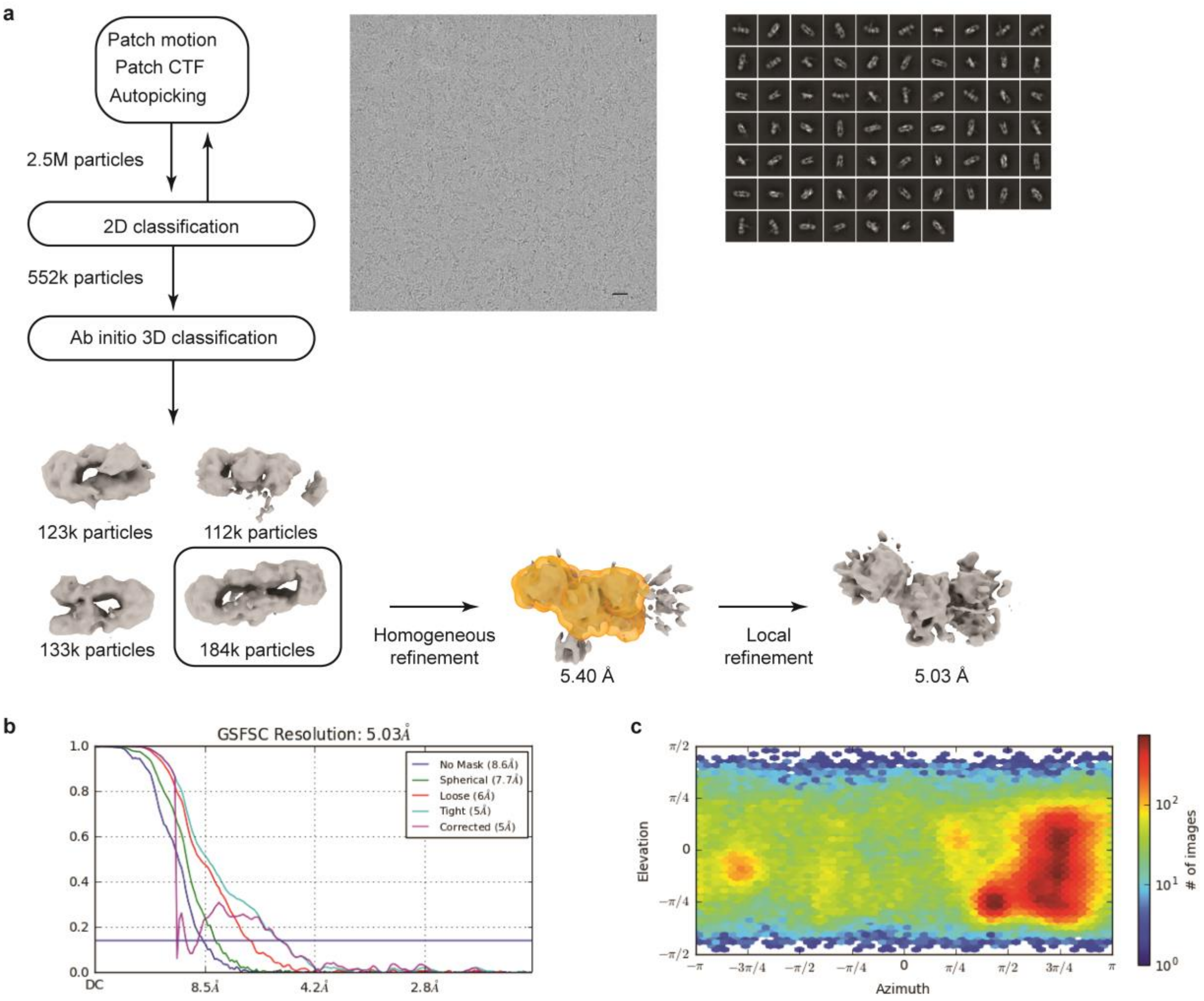

Supplementary Figure 3 | CryoEM 3D reconstruction of the IM459-complexed hIR-A^{ecto}. a, Particle picking, 2D and 3D classification, and 3D reconstruction pipeline, showing on the right a representative micrograph (scale bar = 200 Å) and the selected set of 2D class averages. **b**, Fourier shell correlation statistics. **c**, Angular distribution of particles post refinement. For full details, see Methods.

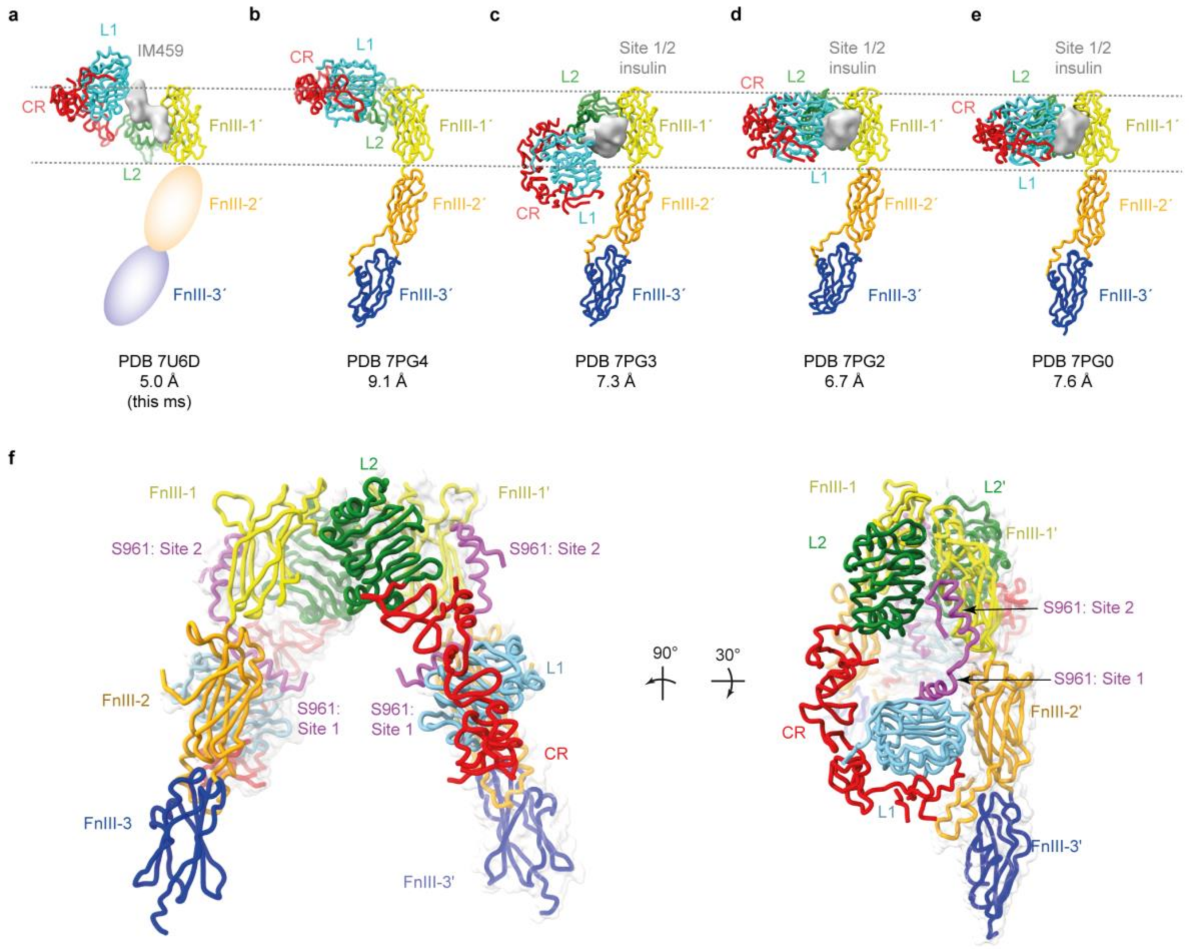

Supplementary Figure 4 | Comparison of the IM459-bound hIR-A^{ecto} with low-resolution insulin-bound hIR structures and proposed mechanism of antagonism by [Site 1]-[Site 2] fusion peptides[3]. a, The IM459-bound IR-A^{ecto} structure reported in this manuscript, showing only a single half of the structure. The IM459 peptide is in grey **b**, The insulin-free half of the two-insulin-bound holo-IR structure corresponding to PDB entry 7PG4[4]. **c**-**e**, The single-insulin-bound of the three-insulin-bound holo-IR structures corresponding to PDB entries 7PG3, 7PG2 and 7PG0, respectively[4]. All five structures are displayed with a common alignment of domains FnIII-1', as indicated by the horizontal dashed lines through each panel. The surfaces depicted in Panels (a), (c), (d) and (e) are low-resolution molecular surfaces derived from the corresponding models of the ligand (grey) within each structure. The Figure illustrates how in (a) the L1-CR module is hinged further away from the membrane than in (c), (d) and (e). In (b), domains L1 and L2 juxtapose, precluding insulin association with domain L1. **f**, Model showing how the receptor could be locked in an apo conformation (PDB 4ZXB)[5,6] by two copies of a [Site 1]-[Site 2] peptide such as the high-affinity antagonist S961 (**Fig. 1c**)[3]. Here, the receptor αCT segments are replaced by the Site 1 component of S961 modelled (based on the known structure of S519C16 bound to the L1-CR module[7]) and with the Site 2 component of S961 modelled according to the complex of IM172N22 presented in this manuscript. In all panels, receptor domains are colored according to the primary structure schematic presented in Supplementary Fig. 2a.

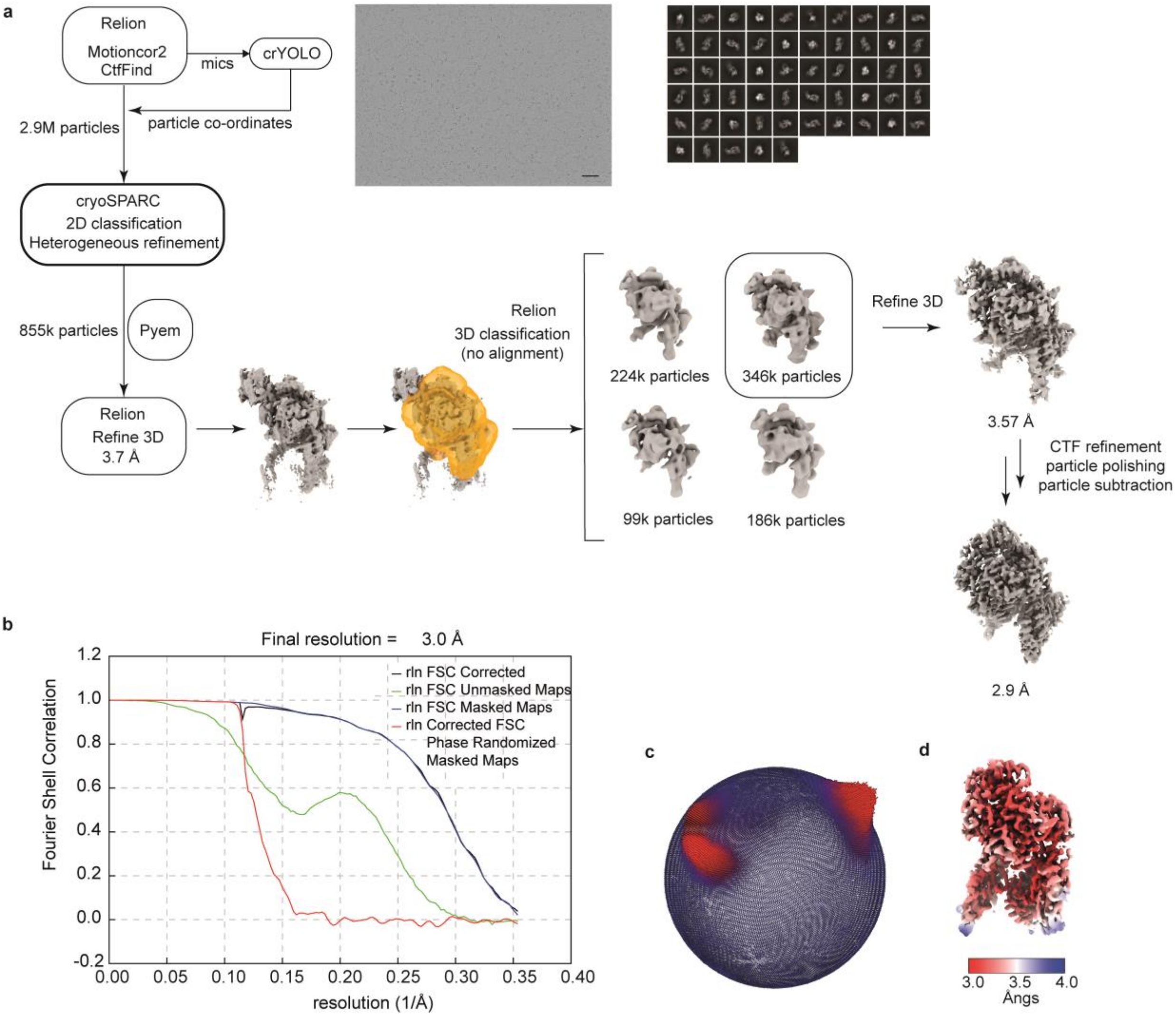

Supplementary Figure 5 | CryoEM 3D reconstruction of the IM172N22-complexed hIRΔβ.zip + hIns + Fv 83-7. a, Particle picking, 2D and 3D classification, and 3D reconstruction pipeline, showing on the right a representative micrograph (scale bar = 400 Å) and the selected set of 2D class averages. **b**, Fourier shell correlation statistics. **c**, Angular distribution of particles post refinement. **d**, Local resolution assessment. For full details, see Methods.

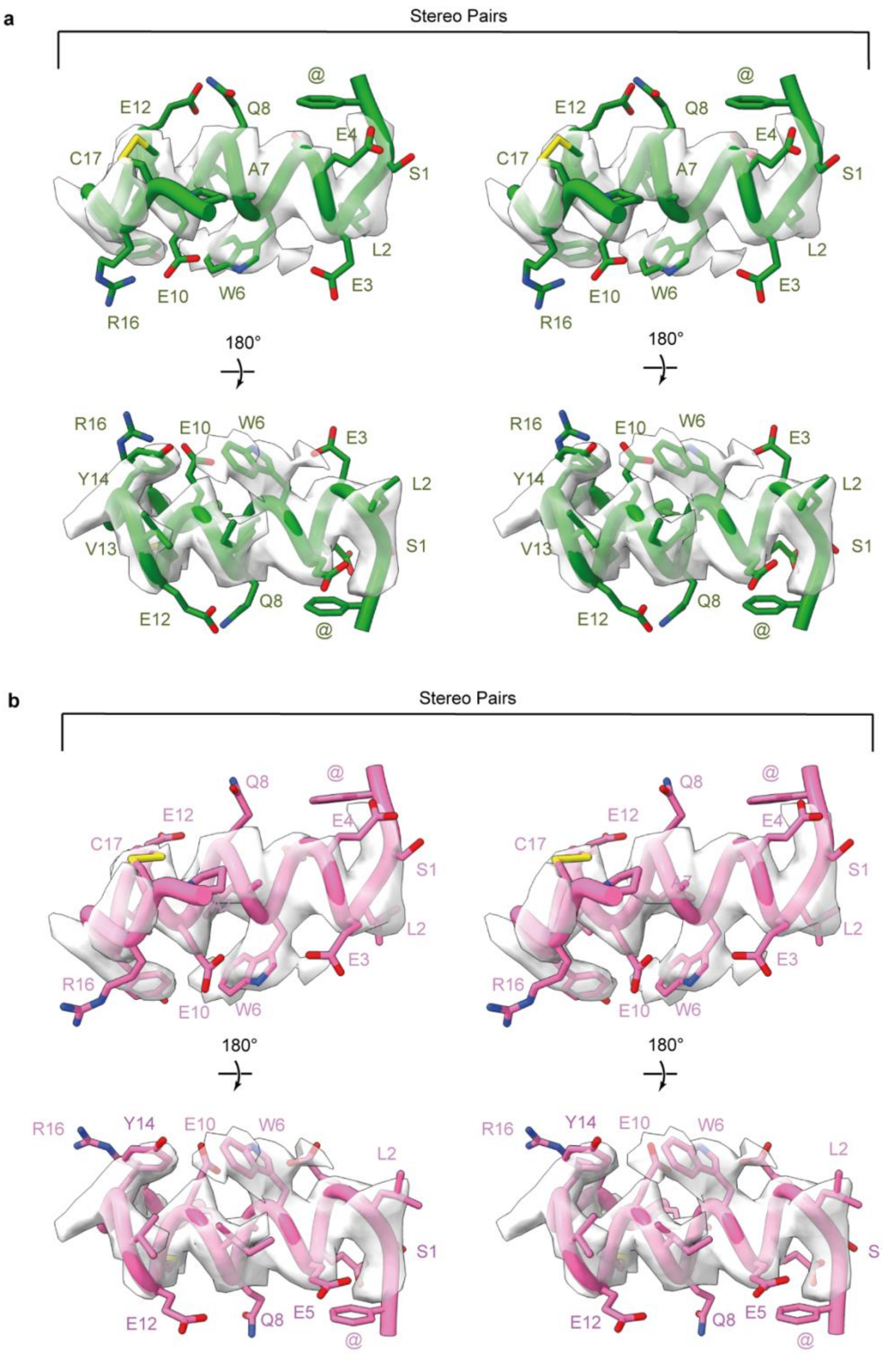

Supplementary Figure 6 | Real-space-averaged cryoEM density associated with IR$\Delta\beta$-zip-bound IM172N22. a, Fit of the IM172N22 moiety (green) located on domain FnIII-1' (white) to averaged density. **b**, Fit of IM172N22 moiety (pink) located on domain FnIII-1 (white) to averaged density. Density in each panel is that within a 3.0 Å "range" of the peptide atoms. Left and right components of each panel are wall-eye stereo views; top and bottom components of each panel are rotated by 180° with respect to each other about a horizontal in-plane-of-paper axis. Selected residues are labelled (@=N-terminal phenylacetylation; see Fig. 1b).

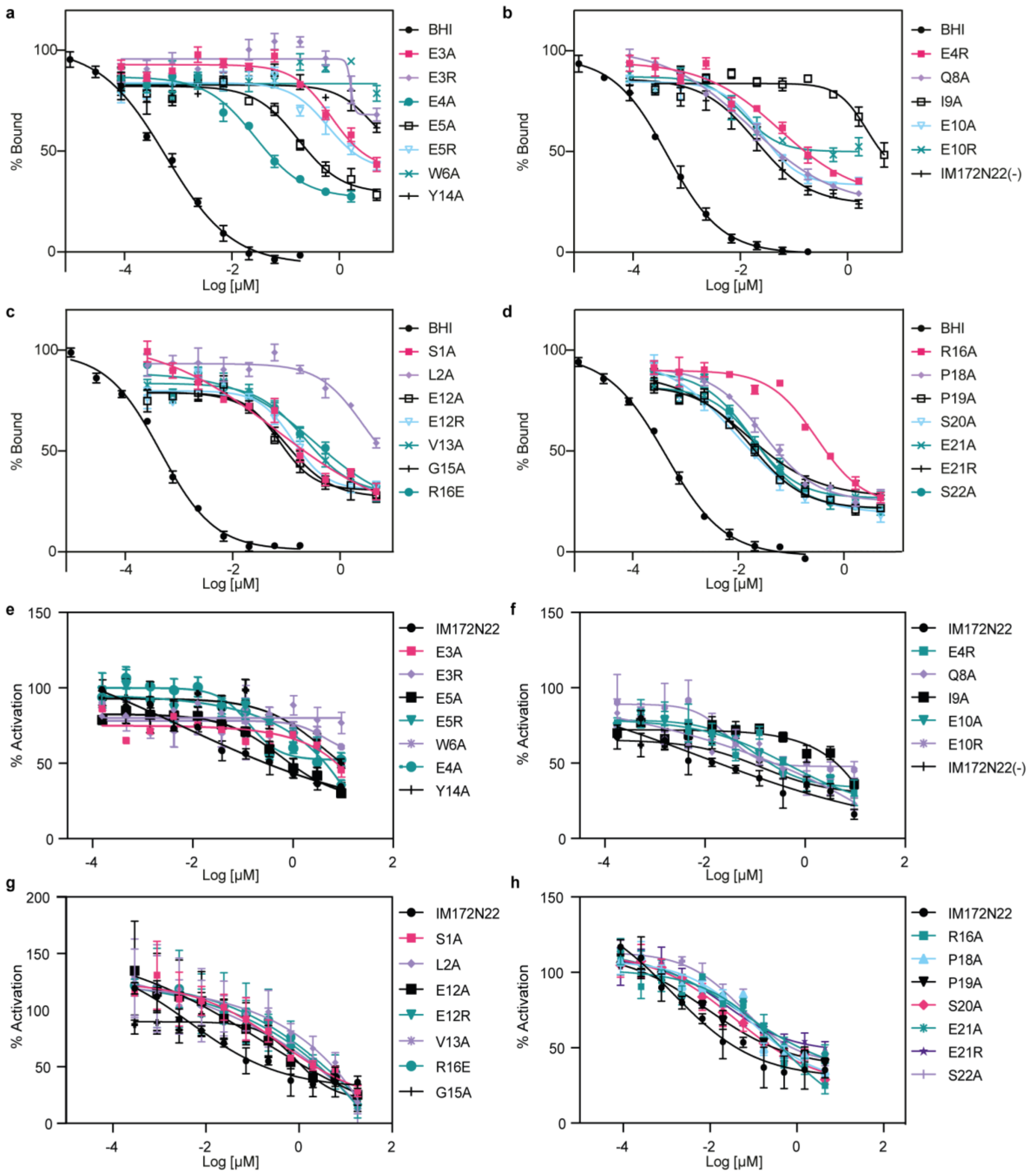

Supplementary Figure 7 | Characterization of mutant IM172N22 peptides. Mutant peptides were partitioned into four subsets for the purpose of the assays. **a-d**, Competition binding assay of the four respective groups of mutant peptides. BHI is a control in each instance. **e-h**, Phosphorylation inhibition assay of the four respective groups of mutant peptides. IM172N22(-) is the native IM172N22 peptide without N-terminal phenylacetylation, all mutant peptides were also synthesized lacked N-terminal phenylacetylation. Error bars represent standard error of the mean for each data point and are obscured by the markers in some instances ($n = 3$ independent replicates, with selected individual measurements deemed aberrant excluded). See Methods for detail. Source data are provided as a Source Data file.

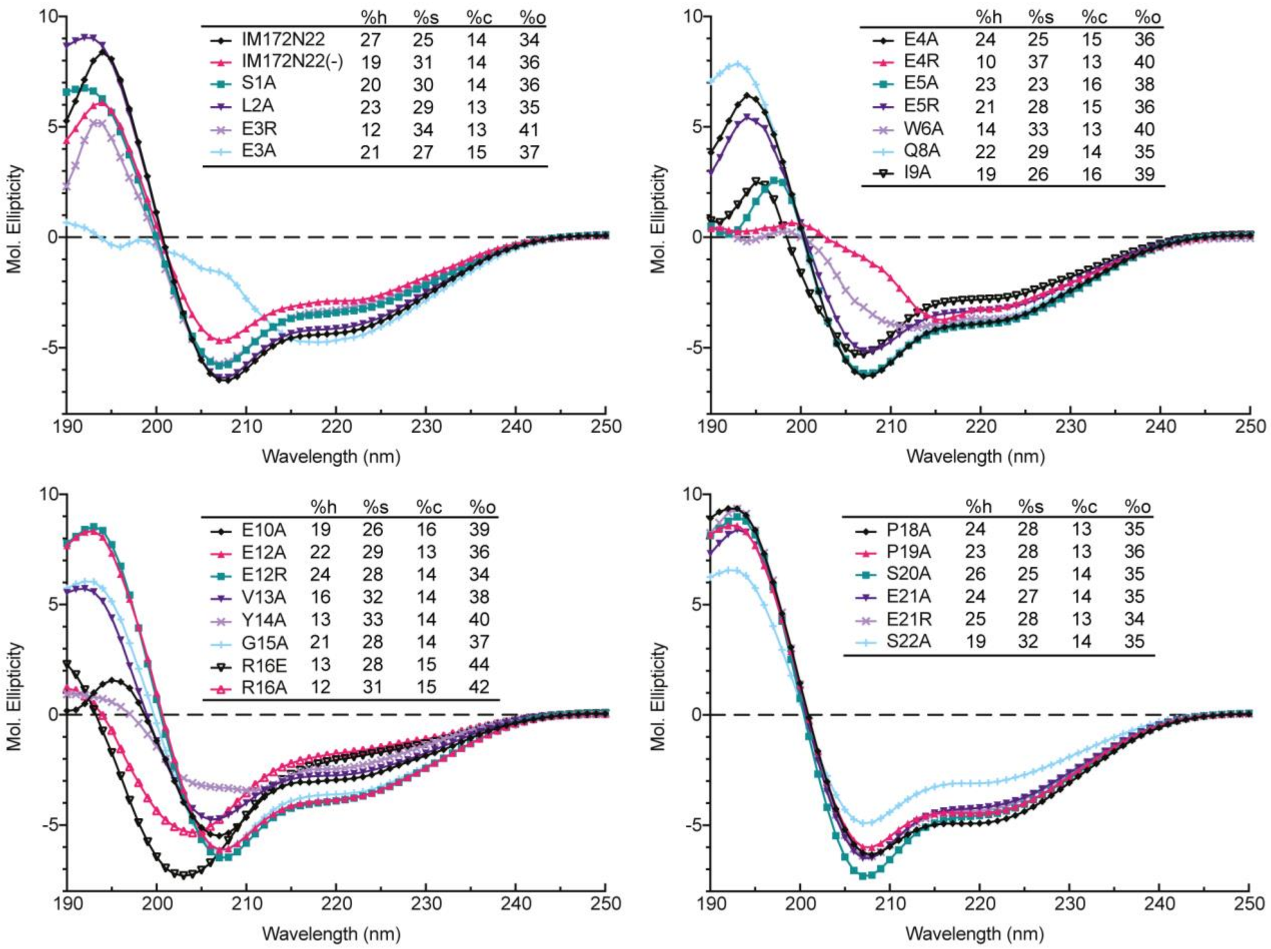

Supplementary Figure 8 | Circular dichroism spectra for IM172N22 mutant peptides. For each peptide, %h, %s, %c and %o represent respectively the calculated percentage of helical, strand, coil and other conformation within the peptide. IM172N22(-) is the native IM172N22 peptide without N-terminal phenylacetylation; all mutant peptides also lack N-terminal phenylacetylation. Source data are provided as a Source Data file.

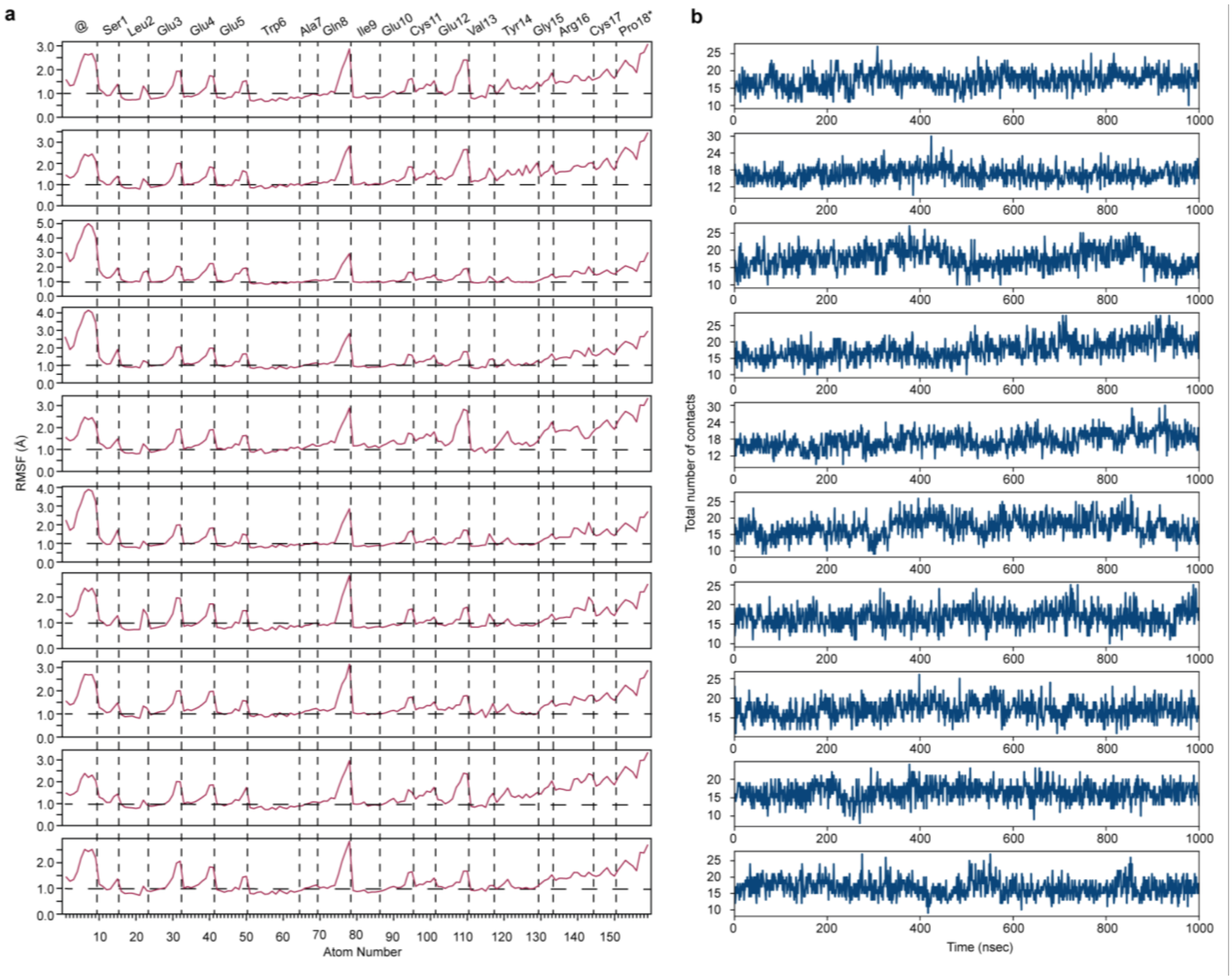

Supplementary Figure 9 | Mobility of IM172N22 during MD simulation. a, The root-mean-square fluctuation (RMSF) of each non-hydrogen atom of the eighteen modelled residues of IM172N22 across ten independent 1.0 μs molecular dynamics simulations. RMSF is defined as

$$\mathrm{RMSF} = \sqrt{\frac{1}{T}\sum_{t=1}^{T}\left(r_i'(t) - r_i(t_{ref})\right)^2}$$

where T = trajectory time, t_{ref} = reference time (the first frame), r = position of atom i in the reference at time t_{ref} and r' = position of atom i at time t after superposition of domain FnIII-1 onto the reference frame. Within each residue, atom numbering increases numerically from backbone through to side chain termini, excluding hydrogens. @ = N-terminal phenylacetyl moiety of residue Ser1. **b**, Plots of the total number of inter-atomic contacts between IM172N22 and hIR domain FnIII-1' during the time course of ten respective independent 1 ns MD simulations.

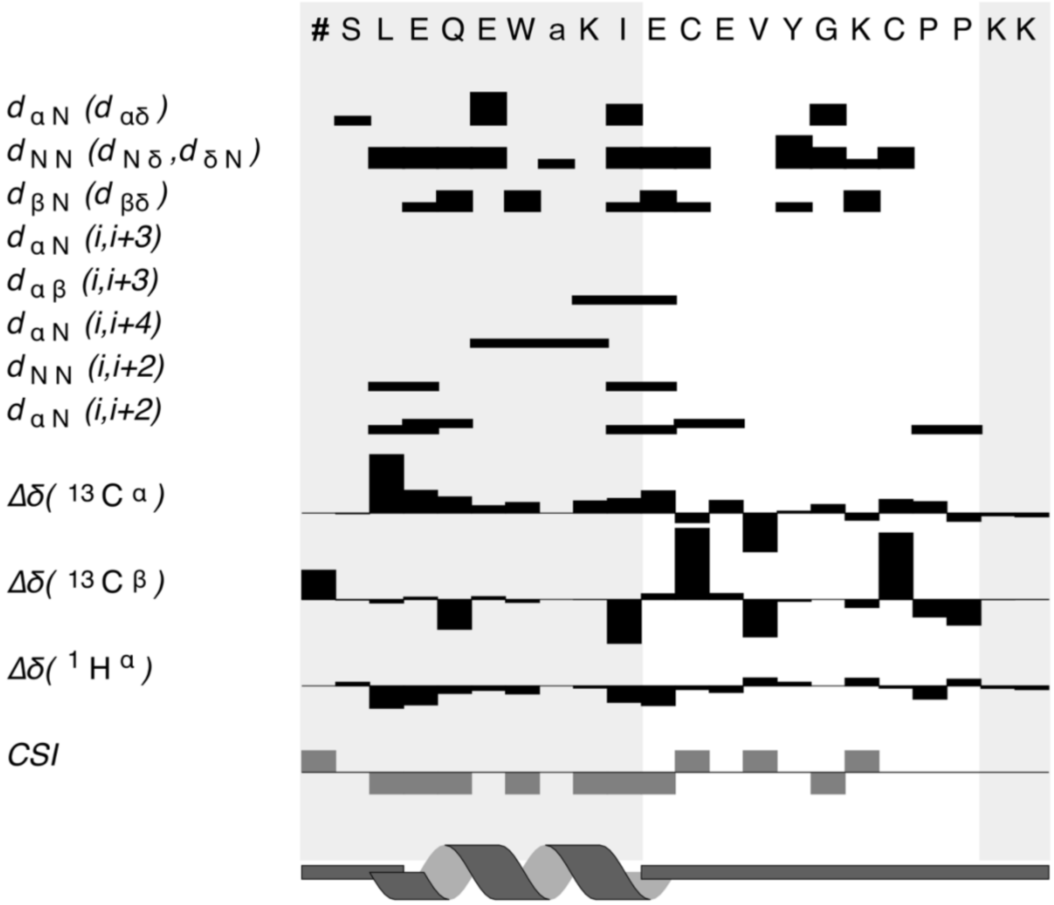

Supplementary Figure 10 | Predicted secondary structure of peptide IM459N21. Secondary structure chart depicting inter-residue NOE cross peaks, chemical shift differences from random coil and chemical shift index and derived from DANGLE dihedral angle estimations [8]. #: N-terminal (4-aminomethyl)phenylacetylation (PDB residue BVK); a: 2-aminoisobutyric acid (PDB residue AIB).

Supplementary Table 1 | Atomic model and cryoEM reconstruction statistics

**Model**	**IM459-complexed IR-A$^{(ecto)}$**		**IM172N22-complexed IRΔβ.zip + Fv 83-7 + insulin**	
PDB ID	7U6D		7U6E	
Composition (#)				
Chains	5		8	
Atoms	7219 (Hydrogens: 0)		8017 (Hydrogens: 0)	
Residues	Protein: 882		Protein: 974	
Water	0		0	
Ligands	NAG: 5		HY1: 2 ; NAG: 8	
Bonds (RMSD)				
Length (Å) (# > 4σ)	0.003 (0)		0.004 (0)	
Angles (°) (# > 4σ)	0.644 (2)		0.839 (0)	
MolProbity score	2.00		1.68	
Clash score	5.82		3.36	
Ramachandran plot (%)				
Outliers	0.12		0.00	
Allowed	6.40		5.87	
Favored	93.48		94.13	
Rama-Z (Ramachandran plot Z-score, RMSD)				
whole (# / Z-score / RMSD)	859 / 3.12 / 0.25		954 / -2.37 / 0.25	
helix (# / Z-score / RMSD)	132 / -2.54 / 0.37		179 / -1.74 / 0.35	
sheet (# / Z-score / RMSD)	131 / -1.33 / 0.44		161 / -1.21 / 0.41	
loop (# / Z-score / RMSD)	596 / -2.28 / 0.22		614 / -1.67 / 0.23	
Rotamer outliers (%)	2.13		1.59	
Cβ outliers (%)	0.00		0.00	
Peptide plane (%)				
Cis proline / general	0.0 / 0.0		2.4 / 0.0	
Twisted proline / general	0.0 / 0.0		0.0 / 0.0	
CαBLAM outliers (%)	2.97		2.24	
ADP (B-factors)				
Iso / Aniso (#)	4953 / 2266		8017 / 0	
Protein (min / max / mean)	192.38 / 925.20 / 411.17		32.91 / 117.62 / 62.92	
Ligand (min / max / mean)	193.57 / 359.71 / 302.69		54.61 / 85.33 / 67.47	
Occupancy				
occ = 1 (%)	100.00		100.00	
**Data**				
Box				
Lengths (Å)	80.56 / 80.56 / 139.92		115.33 / 85.93 / 82.54	
Angles (°)	90.00 / 90.00 / 90.00		90.00 / 90.00 / 90.00	
Supplied Resolution (Å)	6.0		3.0	
Resolution Estimates (Å)	masked	unmasked	masked	unmasked
d FSC (half maps; 0.143)	5.03		3.0	
d 99 (full)	6.5	6.0	3.2	3.1
d model	4.4	4.4	3.1	3.1
d FSC model (0 / 0.143 / 0.5)	4.2 / 5.8 / 9.1	4.3 / 6.2 / 9.4	2.7 / 2.8 / 3.3	2.7 / 2.8 / 3.4
Map (min / max / mean)	-0.11 / 0.40 / 0.03		0.08 / 0.15 / 0.00	
**Model vs. Data**				
CC_{mask}	0.65		0.74	
CC_{box}	0.83		0.80	
CC_{peaks}	0.49		0.71	
CC_{volume}	0.62		0.74	
Mean *CC* for ligands	0.72		0.68	

Supplementary Table 2 | NMR refinement statistics for IM459N21

	**IM459N21**
**NMR distance and dihedral constraints**	
Distance constraints	
Total NOE	223
Intra-residue	116
Inter-residue	
Sequential ($\lvert i-j \rvert = 1$)	65
Medium-range ($1 < \lvert i-j \rvert < 5$)	36
Long-range ($\lvert i-j \rvert \geq 5$)	6
Intermolecular	None
Hydrogen bonds (if any?)	None
Total dihedral angle restraints	28
ϕ	14
ψ	14
**Structure statistics**	
Violations	
Distance constraints (Å) (mean and s.d.)	0.10 ± 0.52
Dihedral angle constraints (°) (mean and s.d.)	16 ± 10
Max. dihedral angle violation (°)	71
Max. distance constraint violation (Å)	2.0
Deviations from idealized geometry	
Bond lengths (Å)	0.011
Bond angles (°)	3.8
Impropers (°)	23
Average pairwise r.m.s. deviation** (Å)	
Heavy	3.30
Backbone	2.25

**Pairwise r.m.s. deviation was calculated among all refined structures (*n*=16).

SUPPLEMENTARY REFERENCES

1. Pillutla, R. *et al.* Insulin and IGF-1 receptor agonists and antagonists. United States patent US 7,7173,005 B2 (2007).
2. Weis, F. *et al.* The signalling conformation of the insulin receptor ectodomain. *Nat. Commun.* **9**, 4420 (2018).
3. Schäffer, L. *et al.* A novel high-affinity peptide antagonist to the insulin receptor. *Biochem Biophys Res Commun* **376**, 380-383 (2008).
4. Nielsen, J. *et al.* Structural investigations of full-length insulin receptor dynamics and signalling. *J. Mol. Biol.*, **434**, 167458 (2022).
5. McKern, N.M. *et al.* Structure of the insulin receptor ectodomain reveals a folded-over conformation. *Nature* **443**, 218-221 (2006).
6. Croll, T.I. *et al.* Higher-Resolution Structure of the Human Insulin Receptor Ectodomain: Multi-Modal Inclusion of the Insert Domain. *Structure* **24**, 469–476 (2016).
7. Lawrence, C.F. *et al.* Insulin Mimetic Peptide Disrupts the Primary Binding Site of the Insulin Receptor. *J. Biol. Chem.* **291**, 15473-15481 (2016).
8. Vranken, W.F. *et al.* The CCPN data model for NMR spectroscopy: development of a software pipeline. *Proteins* **59**, 687-696 (2005).
