# Supplementary material for: Activation of the human insulin receptor by non-insulin-related peptides
Source: Nat Commun. 2022 Sep 28;13:5695. doi: 10.1038/s41467-022-33315-8 (PMC9519552; doi:10.1038/s41467-022-33315-8)
Supplement: Supplementary file 2 — Reporting Summary [file 41467_2022_33315_MOESM2_ESM.pdf]

nature portfolio

Corresponding author(s): Michael Lawrence, David Barrett, Vladislav Kiselyov

Last updated by author(s): Aug 17, 2022

Reporting Summary

Nature Portfolio wishes to improve the reproducibility of the work that we publish. This form provides structure for consistency and transparency in reporting. For further information on Nature Portfolio policies, see our Editorial Policies and the Editorial Policy Checklist.

Statistics

For all statistical analyses, confirm that the following items are present in the figure legend, table legend, main text, or Methods section.

n/a	Confirmed	
☐	☒	The exact sample size (n) for each experimental group/condition, given as a discrete number and unit of measurement
☐	☒	A statement on whether measurements were taken from distinct samples or whether the same sample was measured repeatedly
☒	☐	The statistical test(s) used AND whether they are one- or two-sided<br>*Only common tests should be described solely by name; describe more complex techniques in the Methods section.*
☒	☐	A description of all covariates tested
☒	☐	A description of any assumptions or corrections, such as tests of normality and adjustment for multiple comparisons
☐	☒	A full description of the statistical parameters including central tendency (e.g. means) or other basic estimates (e.g. regression coefficient) AND variation (e.g. standard deviation) or associated estimates of uncertainty (e.g. confidence intervals)
☒	☐	For null hypothesis testing, the test statistic (e.g. F, t, r) with confidence intervals, effect sizes, degrees of freedom and P value noted<br>*Give P values as exact values whenever suitable.*
☒	☐	For Bayesian analysis, information on the choice of priors and Markov chain Monte Carlo settings
☒	☐	For hierarchical and complex designs, identification of the appropriate level for tests and full reporting of outcomes
☒	☐	Estimates of effect sizes (e.g. Cohen's d, Pearson's r), indicating how they were calculated

Our web collection on statistics for biologists contains articles on many of the points above.

Software and code

Policy information about availability of computer code

Data collection	cryoEM: EPU 2
Data analysis	Relion (v 3.1, v 4.0 beta), cryoSPARC (v 2, v 3), crYOLO, ChimeraX (v 1.2.5, v 1.3), ISOLDE (v 1.3.2), PHENIX (v 1.19.2), COOT (v 0.9), NMRPipe (v 10.9 rev 2021.258.11.26), DANGLE within CCPNMR suite (v 2), GROMACS molecular dynamics suite (v 5.0.7), AMBER99SB force field, ACPYPE (beta version), Schrödinger software suite (v 2020.3), Graph Pad Prism (v 9.3.1), Analyzer within Genedata Screener (v 17.0.2).

For manuscripts utilizing custom algorithms or software that are central to the research but not yet described in published literature, software must be made available to editors and reviewers. We strongly encourage code deposition in a community repository (e.g. GitHub). See the Nature Portfolio guidelines for submitting code & software for further information.

Data

Policy information about availability of data

All manuscripts must include a data availability statement. This statement should provide the following information, where applicable:

- Accession codes, unique identifiers, or web links for publicly available datasets
- A description of any restrictions on data availability
- For clinical datasets or third party data, please ensure that the statement adheres to our policy

The study made use of the following publicly available data sets: PDB entries 4ZXB [http://doi.org/10.2210/pdb4ZXB/pdb], 5J3H [http://doi.org/10.2210/pdb5J3H/pdb], 6HN4 [http://doi.org/10.2210/pdb6HN4/pdb], 6HN5 [http://doi.org/10.2210/pdb6HN5/pdb], and 6SOF [http://doi.org/10.2210/pdb6SOF/pdb]. Model

coordinates and cryoEM maps generated in this study have deposited in the PDB and EMDB: IM459-complexed IR-Aecto: PDB code 7U6D [http://doi.org/10.2210/pdb7U6D/pdb], EMDB code EMD-26363 [https://www.ebi.ac.uk/emdb/entry/EMD-26363]; IM172N22-complexed IRΔβ.zip + Fv 83-7 + insulin: PDB code 7U6E [http://doi.org/10.2210/pdb7U6E/pdb], EMDB code EMD-26364 [https://www.ebi.ac.uk/emdb/entry/EMD-26364]; NMR structure of IM459N21: PDB code 8DI2 [http://doi.org/10.2210/pdb8DI2/pdb]. Source data are provided with this paper. Other raw data and biological materials will be made available subject to the negotiation of a suitable agreement setting forth the terms and conditions of transfer and use of any such materials.

Human research participants

Policy information about studies involving human research participants and Sex and Gender in Research.

Reporting on sex and gender	No human participants were involved in the research.
Population characteristics	*Describe the covariate-relevant population characteristics of the human research participants (e.g. age, genotypic information, past and current diagnosis and treatment categories). If you filled out the behavioural & social sciences study design questions and have nothing to add here, write "See above."*
Recruitment	*Describe how participants were recruited. Outline any potential self-selection bias or other biases that may be present and how these are likely to impact results.*
Ethics oversight	*Identify the organization(s) that approved the study protocol.*

Note that full information on the approval of the study protocol must also be provided in the manuscript.

Field-specific reporting

Please select the one below that is the best fit for your research. If you are not sure, read the appropriate sections before making your selection.

☒ Life sciences ☐ Behavioural & social sciences ☐ Ecological, evolutionary & environmental sciences

For a reference copy of the document with all sections, see nature.com/documents/nr-reporting-summary-flat.pdf

Life sciences study design

All studies must disclose on these points even when the disclosure is negative.

Sample size	Assays: A sample size of n = 3 was chosen as this was judged large enough to reveal qualitative differences in Kds, Kis, EC50s and IC50s of the respective ligands. CryoEM : A single respective grid (n = 1) proved sufficient in each instance to provide enough grid squares to create a representative data set of particles
Data exclusions	Assays: Selected measurements were excluded if these were deemed aberrant, see Source Data file. CryoEM: Particles were selected/excluded using standard software and visual approaches.
Replication	Assays: n=3 independent assays were run on three separate days. CryoEM: each data set was derived from a single respective grid.
Randomization	Assays: no randomization was applied, as plate row bias can be picked up during the statistical analysis. CryoEM: n=1 grid, so no randomization applicable.
Blinding	Assays: no blinding was necessary, as the instrumental measurements and subsequent calculations are performed automatically without bias. CryoEM: n=1 grid, so no blinding applicable.

Reporting for specific materials, systems and methods

We require information from authors about some types of materials, experimental systems and methods used in many studies. Here, indicate whether each material, system or method listed is relevant to your study. If you are not sure if a list item applies to your research, read the appropriate section before selecting a response.

Materials & experimental systems

n/a	Involved in the study
☐	☒ Antibodies
☐	☒ Eukaryotic cell lines
☒	☐ Palaeontology and archaeology
☒	☐ Animals and other organisms
☒	☐ Clinical data
☒	☐ Dual use research of concern

Methods

n/a	Involved in the study
☒	☐ ChIP-seq
☒	☐ Flow cytometry
☒	☐ MRI-based neuroimaging

Antibodies

Antibodies used

Rho 1D4: supplied from University of British Columbia 5 December 2017
mAb 83-7: hybridoma supplied by Prof. Ken Siddle (University of Cambridge, UK) and transferred from CSIRO to WEHI under a Technology Transfer Agreement dd January 2007
4G10-HRP: Millipore catalog #16-105, lot #3316045
9E10 and KM5-1C7-8-5: Supplied by WEHI Antibody Facility (WEHI, Australia).

Validation

No explicit antibody validation was undertaken for this project; all the antibodies are in routine use in the authors' institutions.
Original references are:
Rho 1D4: J Biol Chem 1988 v263 p11768-75
83-7: Biochem J 1986 v235 p199-208
9E10: Mol. Cell. Biol. 1985 v5 p3610-6
KM5-1C7-8-5: cited in FEBS Lett 2002 v516 p80-6
4G10-HRP: Routinely evaluated in western blot on RIPA lysates from EGF-treated human A431 carcinoma cells (from manufacturer's web site).

Eukaryotic cell lines

Policy information about cell lines and Sex and Gender in Research

Cell line source(s)

IM-9 human lymphoblast cells: ATCC, #CCL-159
CHO Lec8 cells: ATCC, #CRL-1737
HEK293 cells: ATCC, #CRL-1573
B. choshinensis cells: Takara, Japan

Authentication

No cell line authentication was undertaken for the purposes of this project, the above cells all have established use in the authors' laboratories.

Mycoplasma contamination

Cell lines were tested for and confirmed free of mycoplasma contamination.

Commonly misidentified lines
(See ICLAC register)

None of the above cell lines is within the ICLAC register.
